# Photocatalytic glucose depletion and hydrogen generation for diabetic wound healing

**DOI:** 10.1038/s41467-022-33475-7

**Published:** 2022-09-27

**Authors:** Shengqiang Chen, Yanxia Zhu, Qingqing Xu, Qi Jiang, Danyang Chen, Ting Chen, Xishen Xu, Zhaokui Jin, Qianjun He

**Affiliations:** 1 Guangdong Key Laboratory for Biomedical Measurements and Ultrasound Imaging, School of Biomedical Engineering, Health Science Center, Shenzhen University, Shenzhen, China; 2 Department of Cell Biology and Medical Genetics, School of Basic Medical Sciences, Shenzhen University Health Science Center, Shenzhen, China; 3 Shanghai Key Laboratory of Hydrogen Science & Shanghai Key Laboratory of Hydrogen Science & Center of Hydrogen Science, School of Materials Science and Engineering, Shanghai Jiao Tong University, Shanghai, China; 4 Shenzhen Research Institute, Shanghai Jiao Tong University, Shenzhen, China

**Keywords:** Drug delivery, Photocatalysis, Nanobiotechnology

## Abstract

High-glucose microenvironment in the diabetic foot ulcer (DFU) causes excessive glycation and induces chronic inflammation, leading to the difficulty of DFU healing. Hydrogen-rich water bath can promote the healing of DFU in clinic by virtue of the anti-inflammatory effect of hydrogen molecules, but the long-term daily soaking counts against the formation of a scab and cannot change the high-glucose microenvironment, limiting the outcome of DFU therapy. In this work, photocatalytic therapy of diabetic wound is proposed for sustainable hydrogen generation and local glucose depletion by utilizing glucose in the high-glucose microenvironment as a sacrificial agent. Hydrogen-incorporated titanium oxide nanorods are developed to realize efficient visible light (VIS)-responsive photocatalysis for glucose depletion and hydrogen generation, achieving a high efficacy of diabetic wound healing. Mechanistically, local glucose depletion and hydrogen generation jointly attenuate the apoptosis of skin cells and promote their proliferation and migration by inhibiting the synthesis of advanced glycation end products and the expression of their receptors, respectively. The proposed VIS-photocatalytic strategy provides a solution for facile, safe and efficient treatment of DFU.

## Abstract (web summary)

Hydrogenation is a treatment for chronic inflammation caused by high glucose levels in diabetic ulcers, However, current therapies have limitations. Here, the authors report on the creation of a visible light photocatalytic agent which depletes glucose in the wound and generates hydrogen to aid in diabetic wound healing.

## Introduction

Diabetic foot ulcer (DFU) is one of the commonest chronic complications of diabetes, accompanied by long-term inflammation, dysautonomia and bacterial infection, which make the healing of diabetic wound difficult^1,2^. The high-glucose microenvironment plays an important role in the occurrence and evolution of diabetic wound^3–6^. Typically, the high-glucose microenvironment can promote glycation to cause a series of side effects including chronic inflammation and blood vessel injury^7,8^. Therefore at present, the routine methods for the treatment of diabetic wound are mainly involved in the combination of anti-hyperglycemia, anti-inflammation, the improvement of ischemic hypoxia, and the supplement of tissue nutrients^9–12^. However, the secondary failure of antidiabetic drugs leads to the non-ideal outcomes of diabetic wound treatment, including limited therapeutic efficacy and serious side effects.

Hydrogen molecules have been proven to be a safe and effective anti-inflammatory agent which can ameliorate ischemia-reperfusion injury and activate skin cells to promote wound healing^13–21^. Recently, a hydrogen-producing hydrogel dressing made of living *Bacillus–Chlorella* was developed to light-responsively produce hydrogen for accelerated diabetic wound healing, but the lifetimes of *Bacillus* and *Chlorella* in the dressing were limited (60 h)^22^. In recent years, we have proposed to utilize reductive chemical factors in the microenvironment of diseases such as tumor and arthritis as sacrificial agents of the artificial catalyst to achieve sustainable and controlled hydrogen generation through the photocatalytic route for improved efficacy of hydrogen therapy^23,24^. Near-infrared (NIR) photocatalysis is beneficial to the hydrogen therapy of deep-seated diseases, but the efficiency of photocatalytic hydrogen generation mediated by NIR is generally much lower than that by ultraviolet (UV) and visible (VIS) lights. Compared with NIR-photocatalysis, VIS-photocatalytic hydrogen generation is more suitable for the treatment of superficial diseases such as diabetic wound. Therefore, glucose depletion and hydrogen supply for anti-hyperglycemia and anti-inflammation are highly desired to address the root cause and symptom of diabetic wound, respectively, but their combination has not been reported so far.

**Fig. 1:**
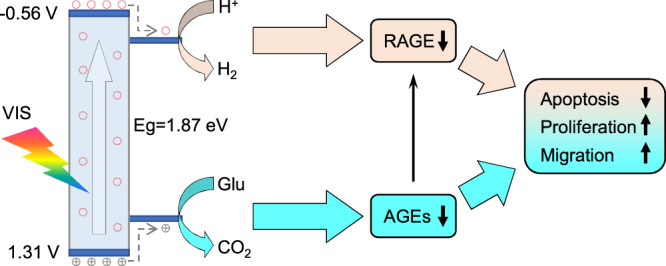
Schematic illustration of the mechanism for visible light-triggered photocatalytic therapy of diabetic wound based on HTON. HTON as a VIS-responsive photocatalyst possesses a right energy band structure which enables to utilize glucose (Glu) in the high-glucose microenvironment as a sacrificial agent for efficient VIS-photocatalytic hydrogen generation. Photocatalytic glucose depletion and generated hydrogen molecules inhibit the synthesis of advanced glycation end products (AGEs) and the expression of their receptors (RAGE) in the microenvironment of diabetic wound, respectively, jointly blocking the apoptosis of skin cells and promoting their proliferation and migration in support of efficient diabetic wound healing.

Here, a kind of hydrogen-incorporated titanium oxide nanorods (HTON) with a rutile single-crystal structure is developed as visible light-sensitive photocatalyst with an appropriate energy band structure to achieve VIS-photocatalytic hydrogen generation by utilizing glucose as sacrificial agent. Glucose oxidation/depletion and hydrogen generation inhibit the glycation process by regulating down the levels of advanced glycation end products (AGEs) and their receptors (RAGE), respectively, jointly blocking the apoptosis of skin cells and inducing their proliferation and migration for improved diabetic wound healing (Fig. 1). Both lowering of systemic blood glucose and foot bathing with hydrogen-rich water hardly bring obvious benefit to DFU in clinic, but the photocatalysis-mediated local glucose depletion and hydrogen generation is able to be competent for significantly accelerated healing of diabetic wound in this work, providing a promising strategy for DFU healing. Moreover, the developed HTON@hydrogel dressing is facile and safe for use, exhibiting a bright prospect of clinical translation.

## Results and discussion

### Morphology, size, and composition characterization of HTON

**Fig. 2:**
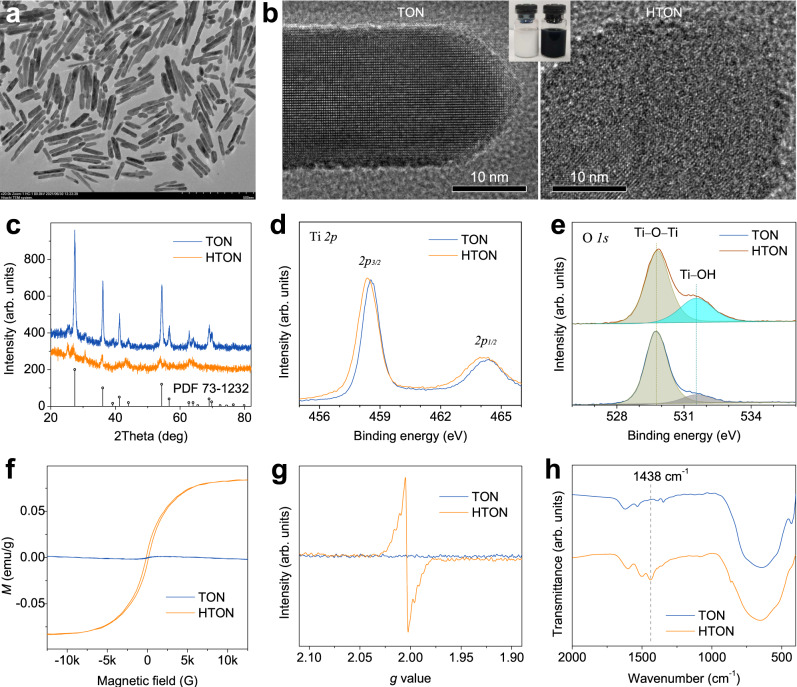
Morphology, composition and size characterization of the HTON nanocatalyst. TEM image of HTON (**a**), HR-TEM images (**b**), XRD (**c**), XPS patterns (**d**, **e**), VSM pattern (**f**), EPR spectra (**g**), and FTIR spectra (**h**) of TON and HTON. The experiments for **a**, **b** were repeated three times independently with similar results.

Titanium dioxide has been approved by the United States Food and Drug Administration (FDA) to be used as an additive in food, medicine and cosmetics owing to high biocompatibility, and its energy band structure is easily tunable by doping to enhance its sensitivity to VIS light and photocatalytic performance. Therefore, doped titanium dioxide was chose for photocatalytic therapy in this work. In order to obtain high aqueous dispersion, titanium dioxide nanorods (TON) were synthesized and incorporated with hydrogen into HTON by a full-solution method. TON with a rutile single-crystal structure was prepared by a hydrothermal method, and then HTON was synthesized by Li incorporation and Li/H exchange in a Li-dissolved ethylenediamine (EDA) solution at room temperature by modifying/combining the previous methods^25–27^. TEM, elementary mapping and dynamic light scattering (DLS) results suggested that as-synthesized THON had a rod-like morphology (about 20 nm in diameter and 100–400 nm in length) and good dispersion (Fig. 2a and Supplementary Figs. 1, 2). High-resolution TEM and XRD results showed that TON was a rutile single crystal (Fig. 2b, c), which was favorable to obtain stable HTON. By comparison, the atomic arrangement of HTON was strongly disordered (Fig. 2b), and the crystalline degree of THON was much worse than that of TON (Fig. 2c), owing to hydrogen incorporation.

X-ray photoelectron spectroscopy (XPS) patterns showed a slight shift of Ti *2p* peaks to lower energies (Fig. 2d and Supplementary Fig. 3), suggesting that hydrogen was successfully doped into TON. Moreover, the O *1* *s* peak of HTON at 531.5 eV was much higher than that of TON (Fig. 2e), meaning the formation of plentiful bridging hydroxyls (Ti–OH–Ti) as the content of Ti−OH bond in Ti−O increased from 18.6% to 30.0% after hydrogen incorporation (Fig. 2e)^26,28^. In addition, Li was not detected in HTON by ICP, and no Li characteristic peak at 55 eV was observable in the XPS pattern (Supplementary Fig. 4), indicating no residue of Li in the as-synthesized HTON. Moreover, the color change from white to black further confirmed successful hydrogen incorporation (Fig. 2b). Furthermore, from vibrating sample magnetometery (VSM) pattern in Fig. 2f, TON and HTON demonstrated typical diamagnetism and paramagnetism, respectively. On the other hand, a strong electron paramagnetic resonance (EPR) signal peak with *g* value of 2.004 was clearly visible in HTON, but not in TON (Fig. 2g). The strong signal overlap between Ti^3+^ and oxygen vacancies (*V*_*o*_) possibly caused the slight shift of EPR peak (2.01 or 2.02 of *g* value for surface Ti^3+^). These results consistently indicated that hydrogen incorporation created a considerable number of Ti^3+^ and *V*_*o*_ defects, facilitating the photocatalytic separation of electrons and holes in favor of photocatalytic hydrogen production^29^. The Raman and FTIR spectra further confirmed the structural change of TON induced by hydrogen incorporation, as suggested by the left-shift and broadening of the *E*_*g*_ vibration mode at 445 cm^−1^ (Supplementary Fig. 5) and the enhanced IR peak at 1438 cm^−1^ (Fig. 2h). In addition, FTIR results showed that there was no residue of EDA and other organic compounds on the as-synthesized HTON (Fig. 2h).

### VIS-photocatalytic performances

**Fig. 3:**
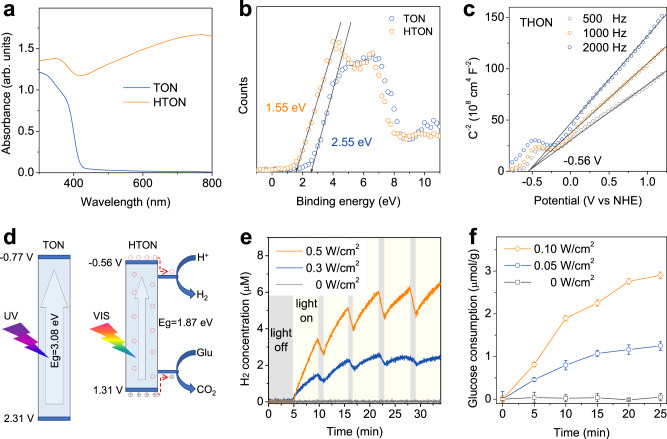
Band structure and VIS-photocatalytic behavior of the HTON nanocatalyst for hydrogen generation and glucose consumption. UV-VIS absorption spectra (**a**), XPS-determined highest occupied molecular orbital (HOMO) levels of TON and HTON (**b**), Mott–Schottky plots of HTON (**c**), band structures (versus normal hydrogen electrode (NHE)) of TON and HTON and the schematic illustration of the mechanism for VIS-photocatalytic hydrogen generation and glucose consumption (**d**), and VIS-photocatalytic hydrogen generation (**e**) and glucose consumption (**f**) behaviors (*n* = 3 biologically independent experiments) of the HTON nanocatalyst in the aqueous solution of glucose (20 mM) under irradiation of xenon lamp at various power densities. Data were presented as mean value ± SD.

Absorption spectra of TON and HTON demonstrated that the white TON only can absorb UV light, but the black HTON exhibited a strong absorbance in the VIS and NIR regions (Fig. 3a), owing to hydrogen incorporation. Moreover, the energy band structure of HTON was detected to check whether HTON was competent for VIS-photocatalytic hydrogen generation and glucose depletion. The valance band (VB) XPS spectra indicated that the VB maximums (*E*_*VBM*_) of TON and HTON were 2.55 eV and 1.55 eV, respectively (Fig. 3b). Then, the VB energy of the corresponding normal hydrogen electrode (*E*_*VB*_ versus NHE) was calculated according to the following formula: *E*_*VB*_ (vs. NHE) = *φ* + *E*_*VBM*_ (vs. XPS) − 4.44, where *φ* was the work function of the instrument (4.20 eV)^30^. Therefore, the HOMO (highest occupied molecular orbital) levels of TON and HTON were determined to be 2.31 and 1.31 V (vs. NHE), respectively. As estimated by the Mott–Schottky curves (Fig. 3c and Supplementary Fig. 6), the flat-band potentials of TON and HTON, which were proximately equal to their LUMO (lowest unoccupied molecular orbital) levels^18^, were calculated to be −0.77 and −0.56 V (vs. NHE), respectively. Correspondingly, the band gaps of TON and HTON were calculated to be 3.08 eV and 1.87 eV, respectively, causing higher VIS-photoelectric activity of HTON compared with TON (Supplementary Fig. 7). The relative band positions of TON and HTON are illustrated in Fig. 3d. It can be found that the band gaps of TON and HTON corresponded to the absorption band edges of 403 nm and 663 nm, respectively, indicating that hydrogen incorporation caused the remarkable red shift of absorption from UV to VIS. Moreover, HTON exhibited distinct localized surface plasmon resonance (LSPR) absorption in the VIS-to-NIR range (Fig. 3a). These two aspects jointly led to the enhanced UV-to-NIR absorption and thus the blackening of HTON (Fig. 2b).

In theory, the LUMO (−0.56 V) and HOMO (1.31 V) levels of HTON are high enough for the evolutions of H^+^-to-H_2_ and glucose-to-gluconic acid (0.45 eV of oxidative potential) (Fig. 3d). As expected, the HTON nanocatalyst can catalyze water splitting for the generation of hydrogen gas in a glucose solution (20 mM) under irradiation of a VIS-emitting xenon lamp (400–700 nm), and the hydrogen generation rate depended on the Xenon lamp power density, as higher power density caused more hydrogen production (Fig. 3e). Moreover, hydrogen generation was highly controllable by switching on/off lamp (Fig. 3e). When VIS irradiation was switched off, hydrogen concentration went down a little bit, possibly because of low solubility and easy escape of hydrogen gas from the solution. Simultaneously, glucose concentration was also monitored by a glucose kit. It can be found that glucose consumption was dependent on xenon lamp irradiation (Fig. 3f). No VIS irradiation did not cause glucose consumption, while both higher power density and longer time duration of VIS irradiation resulted in more glucose consumption (Fig. 3f). Therefore, both hydrogen generation and glucose consumption can be controlled facilely in favor of glucose depletion and anti-inflammation at the site of HTON-covered diabetic wound.

### In vitro photocatalytic therapeutic effects and mechanisms

**Fig. 4:**
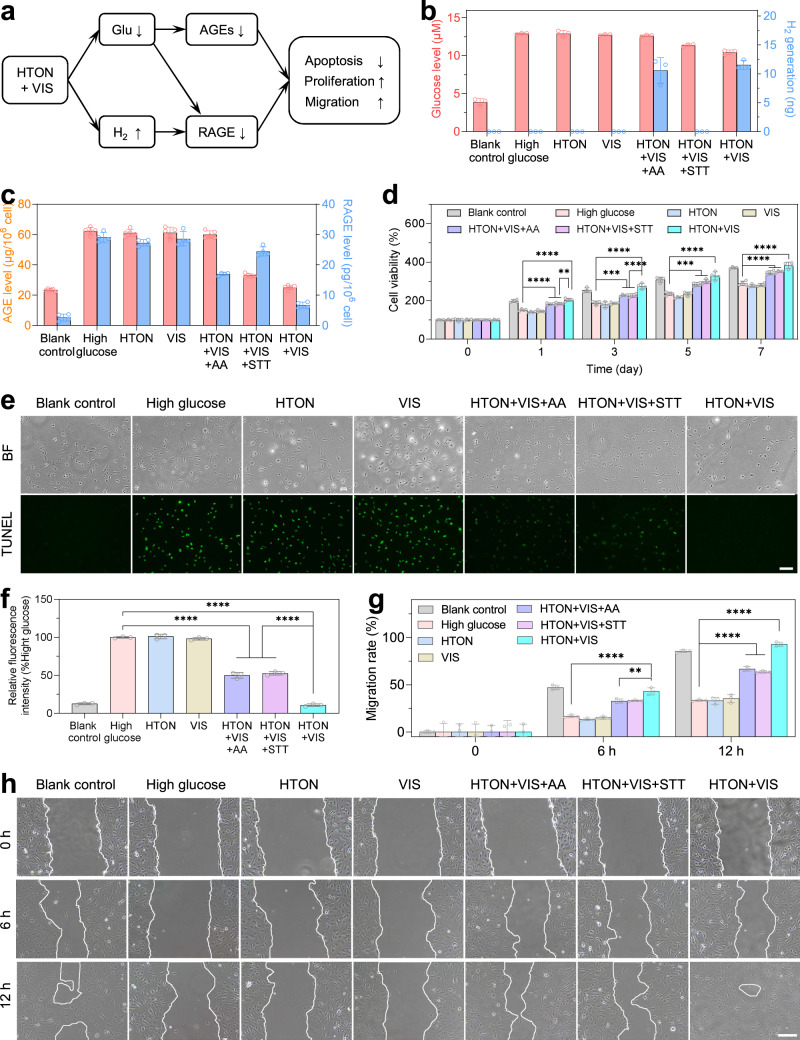
In vitro effects and mechanisms of photocatalytic therapy based on glucose depletion and hydrogen generation. The schematic illustration of the mechanisms for VIS-photocatalytic therapy of diabetic wound (**a**), the intracellular VIS-photocatalytic H_2_ generation and glucose depletion (*n* = 3 biologically independent samples) (**b**), and the expression of advanced glycation end products (AGEs) (*n* = 5 biologically independent samples) and their receptors (RAGE) (*n* = 4 biologically independent samples) levels after 3 days of in vitro cell culture (**c**), the effects of VIS-photocatalytic therapy on the proliferation (*n* = 4 biologically independent samples) (**d**), apoptosis (*n* = 3 biologically independent samples) (**e**, **f**) and migration (*n* = 3 biologically independent samples) (**g**, **h**) of HMEC-1 cells. Fluorescein labeled deoxyuridine triphosphate (dUTP-FITC) from the TUNEL (terminal-deoxynucleoitidyl transferase mediated dUTP nick end labeling) kit was used for green fluorescence imaging of apoptotic cells in **e**. The cellular migration after various treatments for different time periods was identified by drawing the white lines at the edge of cells in **h**. *L*-ascorbic acid (AA) and sodium tetrathionate (STT) were used as hole-sacrificial and electron-sacrificial agents for individual hydrogen generation and glucose depletion, respectively. Scale bars in **e**, **h** correspond to 100 μm and 400 μm, respectively. *P* values were calculated by the one-way ANOVA method. **d** **P = 0.0019; ****P < 0.0001 for Day 1; ***P = 0.0003; ****P < 0.0001 for Day 3; ***P = 0.0006; ****P < 0.0001 for Day 5; ****P < 0.0001 for Day 7. **f** ****P < 0.0001. **g** **P = 0.0011, ****P < 0.0001. Data were presented as mean value ± SD. The experiments for (**e**, **h**) were repeated three times independently with similar results.

It is well known that high-glucose enhances the glycation characterized by increased tissue AGEs, which prevent diabetic wound from healing by inducing the apoptosis of skin cells and inhibiting their proliferation and migration^7,8^. It has been well proved that glucose depletion can inhibit the formation of AGEs^31,32^. Therefore, we speculated that VIS-photocatalatic glucose depletion could bring benefits for diabetic wound healing (Fig. 4a).

At first, we checked the VIS-photocatalytic glucose depletion and hydrogen generation in cells. From Fig. 4b, the VIS-photocatalysis with HTON + VIS (200 μg/mL, 0.1 W/cm^2^, 3 min) can effectively deplete excessive glucose and simultaneously generate H_2_ in HMEC-1 cells, and the HTON + VIS + AA (*L*-ascorbic acid, as hole-sacrificial agent with a redox potential of 0.066 V which is lower than the VB of HTON^33^, at the safe concentration of 250 μg/mL, Supplementary Fig. 8) and HTON + VIS + STT (sodium tetrathionate, as electron-sacrificial agent with strong oxidability^23^, at the safe concentration of 250 μg/mL, Supplementary Fig. 8) treatments can be competent for the controls of individual hydrogen generation and individual glucose depletion, respectively. Correspondingly, glucose depletion with HTON + VIS + STT and HTON + VIS caused the visible decrease of the AGEs levels inside and outside three kinds of typical skin cell lines, human immortalized keratinocyte (HaCaT cells), human skin fibroblast (HSF cells) and human microvascular endothelial cells (HMEC-1 cells) to the similar extent, but other groups did not (Fig. 4c and Supplementary Figs. 9–11).

Besides glucose depletion, the role of VIS-photocatalytically generated H_2_ was recognized by using the RNAseq technique to screen differentially-expressed genes in HSF cells before and after treatment with hydrogen molecules. It can be found that hydrogen molecules induced the up-regulation and down-regulation of 198 and 228 genes in HSF cells, respectively, compared with blank control (Supplementary Fig. 12). It is worth noting that H_2_ treatment remarkably inhibited the AGE-RAGE pathway in the diabetic wound (Supplementary Fig. 13) by regulating down RAGE genes (Supplementary Fig. 14) in accordance with the polymerase chain reaction (PCR) measurement results (Supplementary Fig. 15). Furthermore, hydrogen generation with HTON + VIS + AA and HTON + VIS remarkably inhibited the expression of intracellular RAGE level, but the HTON and VIS groups did not (Fig. 4c). In addition, glucose depletion with HTON + VIS + STT also made a slight contribution to the inhibition of RAGE expression (Fig. 4c), possibly owing to the feedback from the glucose depletion-induced decrease of AGEs in the AGE-RAGE pathway^34,35^. From above results, we can conclude that the VIS-photocatalytic therapy with HTON + VIS efficiently blocked the glycation pathway by simultaneously reducing intracellular production of AGEs and RAGE (Fig. 4a).

Since the glycation pathway in diabetic wound severely affects the apoptosis, proliferation and migration of skin cells to prevent from wound healing^3–8,36^, we further investigated the outcomes of in vitro VIS-photocatalytic therapy with HTON + VIS using three kinds of typical skin cell lines, HaCaT cells, HSF cells and HMEC-1 cells. Firstly, to study the apoptosis model induced by high glucose, cells were cultured with different concentrations of glucose for 3 days, and then stained with dUTP-FITC (fluorescein labeled deoxyuridine triphosphate) from a TUNEL (terminal-deoxynucleoitidyl transferase mediated dUTP nick end labeling) kit for green fluorescence imaging of apoptotic cells. It was found that at least 75, 75, and 25 mM of glucose concentration induced the distinct apoptosis of HaCaT, HSF and HMEC-1 cells, respectively (Supplementary Fig. 16), and therefore used for the following investigation about apoptosis. In high glucose, HTON exhibited high cytocompatibility without obvious toxicity to these three types of cells at the concentration of 0–200 μg/mL (Supplementary Fig. 17), and therefore we used 200 μg/mL of HTON for the following cell experiments.

We confirmed that high glucose (25, 75 and 75 mM) can indeed clearly induce the apoptosis of HMEC-1 (Fig. 4e, f), HaCaT and HSF cells (Supplementary Figs. 16, 18), and inhibit their proliferation (Fig. 4d, Supplementary Fig. 19) and migration (Fig. 4g, h, Supplementary Figs. 20, 21), which was not affected by the controls of individual HTON and VIS (Fig. 4d–h and Supplementary Figs. 18–21). It was thought that the effect of high glucose on these cellular behaviors might derive from glycation-induced oxidative stress^3,37^. Both hydrogen production (HTON + VIS + AA) and glucose depletion (HTON + VIS + STT) can make partial contributions to the apoptotic attenuation and proliferative recovery of the investigated cells, causing a synergetic effect of photocatalytic therapy with HTON + VIS (combined therapy group) (Fig. 4d–h), possibly owing to combined inhibition to the glycation pathway (Fig. 4a). Interestingly, both glucose depletion (HTON + VIS + STT) and hydrogen generation (HTON + VIS + AA) can neutralize the anti-migration effect of high glucose to a certain extent, but photocatalytic therapy with HTON + VIS can even promote the migration of HaCaT cells hugely combined with blank control (Fig. 4g, h, Supplementary Figs. 20, 21), possibly owing to the skin cells migration-promoting effect of hydrogen molecules. Summarily, the anti-apoptosis, pro-proliferation and pro-migration effects of photocatalytic glucose depletion and hydrogen production would greatly favor diabetic wound healing.

### In vivo photocatalytic therapy outcomes

**Fig. 5:**
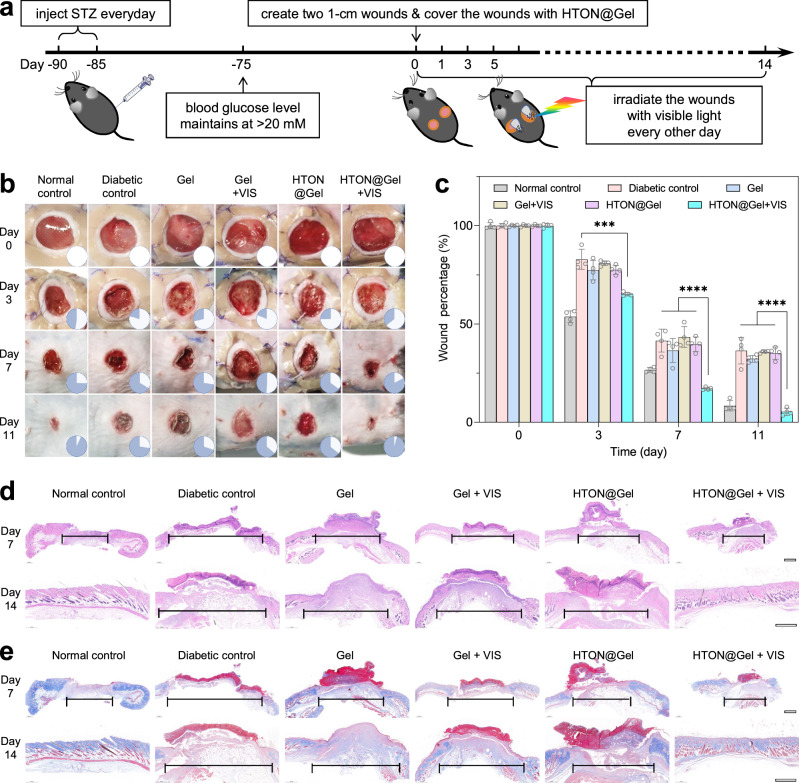
In vivo performances of photocatalytic therapy with HTON. The diabetic wound model building and the treatment procedure with HTON@Gel (**a**), the digital images of diabetic wounds (**b**) and the corresponding wound size statistic (**c**) during treatment (*n* = 5 biologically independent samples), hematoxylin-eosin (H&E) (**d**) and Masson’s trichrome (**e**) staining images of wound tissues extracted at day 7 and day 14. Scale bars in **d**, **e**, 1000 μm. *P* values were calculated by the one-way ANOVA method (***P = 0.003; ****P < 0.0001). Data were presented as mean value ± SD. The experiments for **d**, **e** were repeated three times independently with similar results.

The in vivo photocatalytic therapy efficacy of HTON was evaluated on a full-thickness diabetic wound model (Fig. 5a). The diabetic wound model was established on C57BL/6 mice via intraperitoneal injection of streptozotocin (STZ) for 5 consecutive days, as the STZ-induced diabetic animal model has been widely used to investigate diabetic wound healing^38,39^. The mice, whose blood glucose level substantially went over 20 mM after STZ injection for 10 days and then maintained for 75 days in order to simulate the pathological environment of long-term hyperglycemia in diabetic foot patients, were applied for diabetic wound establishment and then treatment. Full-thickness dorsal skin punch wounds with 10 mm in diameter were created with a round punch and fixed with a circular ring, followed by treatment. The chitosan/hyaluronate gel (Gel) was employed as a carrier of HTON based on high biocompatibility (Supplementary Fig. 22) and temperature sensitivity for convenience of preparation and dressing on the wound. In order to balance the transmittance and photocatalytic hydrogen-generating efficiency of the HTON@Gel dressing, 0.6 mg/mL of HTON loading capacity was chose to obtain a considerable transmittance of 85%. The animals were randomly divided into five groups: diabetic control, Gel, Gel + VIS (0.05 W/cm^2^, 15 min VIS irradiation every other day), HTON@Gel (0.6 mg HTON per mL Gel), and HTON@Gel + VIS (0.6 mg/mL, 0.05 W/cm^2^, 15 min VIS irradiation every other day), and a normal wound without diabetes was used as a normal control group.

From representative pictures of wounds during treatment in Fig. 5b, c, the wound closure rate in the HTON@Gel + VIS group was remarkably higher than that in the other groups, and closed to that in the normal control group without diabetes, suggesting that HTON-mediated VIS-photocatalytic glucose depletion and hydrogen generation played an important role to accelerate diabetic wound healing. By comparison, the contributions of Gel, HTON and VIS were quite limited. Furthermore, the functions of major organs (heart, liver, spleen, lung and kidney) and blood system were not injured, indicating high biocompatibility of HTON@Gel and the used VIS light (Supplementary Figs. 23–25). In addition, the diabetic mice exhibited typical diabetic complications, including the diffuse steatosis and vacuolar degeneration of the liver, the narrowing of hepatic sinusoids and the disordered arrangement of hepatic cords, which were not significantly improved or worsened by various treatment groups. Noticeably, the body weights of mice in the HTON@Gel+VIS group and normal mice without diabetes more quickly recovered and then much more increased over time compared with the other groups, suggesting the visible benefit from the wound repair effect of VIS-photocatalytic therapy (Supplementary Fig. 26).

Furthermore, the evolution of the microscopic structure of new skins during wound healing was investigated by using the hematoxylin-eosin (H&E) and Masson’s trichrome staining methods. From Fig. 5d, e, the inflammatory response in the HTON@Gel + VIS group was significantly reduced and the wounds were filled with granulation tissue after 14 days. In contrast, obvious inflammation and edema were still observable in the other diabetic groups, causing the loss of skin structure and major function as most of natural diabetic wound healing generally does. It was worth noting that there were a large number of living cells including endothelial cells, fibroblasts and keratinocytes in the wound bed after 14-day treatment with HTON@Gel+VIS, suggesting that VIS-photocatalytic therapy successfully promoted the proliferation and migration of skin cells at the diabetic wound site in accordance with the in vitro results (Supplementary Figs. 20–22).

**Fig. 6:**
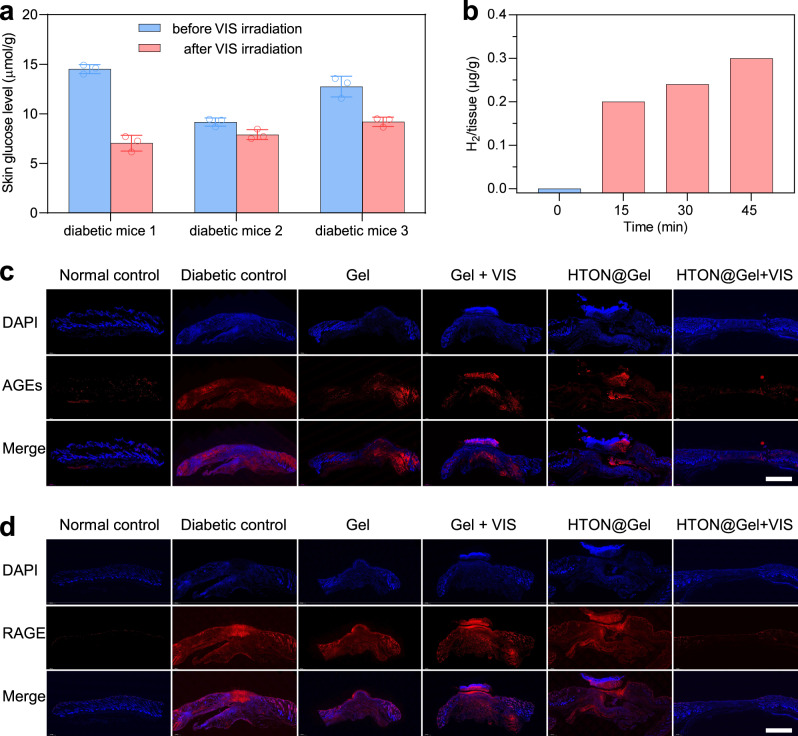
The effect of HTON-mediated photocatalytic therapy on in vivo AGEs and RAGE expression at the site of diabetic wound. The concentrations of glucose (*n* = 3 biologically independent samples) (**a**) and H_2_ (**b**) before and after VIS irradiation at the site of diabetic wound, and immunofluorescence images of wound tissues extracted at day 14 for determining the expression of advanced glycation end products (AGEs) (*n* = 5 biologically independent samples) (**c**) and their receptors (RAGE) (**d**) with and without VIS-photocatalytic therapy. Data were presented as mean value ± SD. Scale bars in **c**, **d**, 1000 μm. The experiments for **c**, **d** were repeated three times independently with similar results.

To further confirm the proposed mechanism for VIS-photocatalytic therapy of diabetic wound in vivo, we detected the VIS-photocatalytic hydrogen generation and glucose depletion at the site of HTON@Gel + VIS treated diabetic wound using gas chromatography (GC) and a glucose kit, and photoluminescence and immunofluorescence assays were also performed to examine the expression of AGEs and RAGE at the wound site. It can be found that the glucose concentration at the site of diabetic wound remarkably decreased after HTON@Gel + VIS treatment (0.05 W/cm^2^, 15 min) (Fig. 6a), and H_2_ was simultaneously generated there, whose yield was dependent on VIS irradiation time (Fig. 6b). But systemic blood glucose levels were not reduced by local photocatalytic glucose depletion in the present treatment conditions (Supplementary Fig. 27). However, it is possible that oral administration of hypoglycemic drugs can further enhance the photocatalytic therapeutic outcomes. Importantly, both AGEs and RAGE were distinctly over-expressed in the diabetic wound during evolution (Fig. 6c, d and Supplementary Figs. 28–30), which was proved to be one of major reasons of blocked healing^7,8^. The Gel, Gel + VIS and HTON@Gel groups made slight and similar contributions to the reduced expression of AGEs (Supplementary Figs. 28, 29), possibly owing to the repair effect of Gel (the CS/HA gel). By comparison, the VIS-photocatalytic therapy with HTON@Gel + VIS more remarkably depressed the levels of both AGEs and RAGE in the diabetic wound in accordance with the in vitro results, which was attributed to locally photocatalytic glucose depletion and hydrogen generation (Fig. 4).

Furthermore, we investigated the effect of photocatalytic therapy on the diabetic wound microenvironment. The depressed expressions of CD31 and VEGF in the diabetic model were sharply recovered, especially at the early stage (Day 7, Supplementary Fig. 31), due to the anti-apoptosis, pro-proliferation and pro-migration effects of photocatalytic hydrogen generation and glucose depletion based on HTON (Fig. 4). This indicated that the photocatalytic inhibition of the AGE-RAGE pathway promoted angiogenesis in support of diabetic wound healing.

In summary, we designed and synthesized a kind of HTON with a rutile single-crystal structure by the full-solution incorporation methods. The modulation of band structure by hydrogen incorporation endowed HTON with visible light absorption and VIS-photocatalytic activity, and enabled glucose in the high-glucose microenvironment of diabetic wound to be used as sacrificial agent. HTON-mediated VIS-photocatalysis to realize the controlled and sustainable glucose depletion and hydrogen generation in vitro and in vivo, which together attenuated the pro-apoptotic effect of high glucose, and promoted the cellular proliferation and migration in support of diabetic wound healing. Overall, both excellent diabetic wound healing performance and high biosafety of HTON plus visible light ensured a high potential for clinical translation.

## Methods

### Synthesis of HTON and the HTON@Gel dressing

TON with the rutile single-crystal structure was prepared by the hydrothermal method. 3.8 g TiCl_4_ was slowly added into 20 mL ice water and continuously stirred for 30 min at a speed of 500 r/min. Then the solution was filtered to get rid of potential insoluble species, and put into a 50 mL Teflon-lined stainless steel autoclave to conduct the hydrothermal reaction at 220 °C (heating rate of 10 °C/min). After reaction for 2 h, the autoclave was naturally cooled to room temperature, and then the white precipitate was collected by centrifugation (15,000 × *g*), washed three times with anhydrous ethylenediamine (EDA) and finally dispersed in anhydrous EDA. Argon was used to replace other gases in the solution by the upper exhaust method for the following reaction.

140 mg lithium metal foil was dissolved in 16 mL EDA to form a solvated electron solution, and 4 mL EDA solution of TON (50 mg/mL) was added. The solution was stirred for 6 days in closed and anhydrous conditions. After sufficient reaction, 50 mL HCl (1 mol/L) was slowly dropped into the mixture to quench the excessive electrons. Finally, HTON was collected by centrifugation (15,000 × *g*) and then rinsed with deionized water several times.

For the construction of hydrogel dressing with HTON, 2% chitosan (CS), 10% *β*-glycerophosphate (GP) and 1% sodium hyaluronate (HA) were prepared in 0.1 M hydrochloric acid, deionized water and deionized water, respectively, and subsequently mixed at a volume proportion of 5:3:2, and then HTON was dispersed into the mixed solution at a loading capacity of 0.6 mg/mL under assistance of ultrasound and shaking. As to treatment on the animal model, the freshly prepared HTON@Gel was immediately applied onto the wound so that it can be naturally gelled on the wound by body heat.

### Characterization of TON and HTON

The morphology and size of TON and HTON were measured by TEM (HT7700, HITACH, and JEM-200F). The hydrodynamic size of nanorods was measured on a Malvern Zetasizer Nano ZS90. X-ray diffraction (XRD) patterns were recorded on a D8 Advance diffractometer (Germany Brock AXS Company, Cu Kα, λ = 1.54056 Å) operated at 40 kV and 200 mA at room temperature. The UV absorption spectra were recorded on the UV-Vis spectrophotometer (UV-3600 PLUS, Shimadzu). The composition of TON and HTON were characterized by Fourier transform infrared spectroscopy (FTIR) on a Thermo-Nicolet Nexus 670A TR-IR spectrometer. The X-ray photoelectron spectroscopy (XPS) was measured on a Thermo Scientific Kα photoelectron spectroscopy with Al Kα radiation. Raman spectra were measured on a Raman imaging microscope (Thermos scientific, DXR3xi, 532 nm Filter). EPR spectra were measured on a Bruker EMXnano spectrometer at 9.44 GHz and 300 K. VSM patterns were collected on a LakeShore7404 spectrometer at 300 K. The Mott–Schottky plots of nanorods-coated indium tin oxide (ITO) glass were measured on a CHI760D electrochemical station (Shanghai Chenhua, China). A Xe lamp with a 400 nm cutoff filter was utilized as the visible light source for VIS-photocatalytic therapy. During the Mott–Schottky test, a standard three-electrode device with the ITO glass as the working electrode, the platinum foil as the counter electrode, and the Ag/AgCl electrode as the reference electrode were used. Three electrodes were inserted into the quartz battery pack and the Mott–Schottky plots were measured in 0.5 M Na_2_SO_4_ electrolyte.

### Detection of hydrogen generation and glucose depletion

In the simulated solution, the hydrogen electrode (Unisense, Denmark) was used to detect the VIS-photocatalytically generated hydrogen over HTON under irradiation of the Xe lamp. The simulated high-glucose environment was prepared by adding HTON into the glucose solution (20 mM) and the final concentration of HTON was set to be 200 μg/mL. Briefly, 1 mL of the prepared solution was put into a special container, and then the hydrogen electrode was placed above the solution to detect hydrogen molecules by repeatedly switching on/off the Xe lamp which irradiated on the solution at fixed time points in the dark. The glucose concentration at the corresponding time points were determined using a glucose assay kit (Solarbio, BC2505) after the collected solution was diluted ten folds. In vitro, the cellular supernatant was collected to detect hydrogen concentration after VIS irradiation, and meanwhile the cellular supernatant was filtered to remove nanoparticles followed by detection of the glucose concentration using the glucose assay kit (Solarbio, BC2505). In vivo, after VIS irradiation (0.05 W/cm^2^) for fixed time duration (15, 30 or 45 min) on the HTON@Gel covered diabetic wound, the skin tissue of wound was immediately collected, and placed in a 2 mL homogenization tube followed by sealing with a rubber cap. After homogenization in the PBS buffer for 90 s, a GC sampling needle was used to absorb the gas in the sealed homogenization tube for the GC measurement of hydrogen generation. On the other hand, the homogenized tissue solution was centrifugated (15,000 × *g*), and the supernatant was collected to measure the glucose concentration in the treated skin by using the glucose assay kit.

### Measurement of high glucose induced apoptosis and photocatalytic anti-apoptosis

HaCaT, HSF and HMEC-1 cell lines were purchased from Procell Life Science & Technology Co., Ltd. (Wuhan, China). HaCaT and HSF cells were cultured with Dulbecco’s modified Eagle’s medium (DMEM, Gibco, USA) containing 10% fetal bovine serum (FBS, Gibco, USA), while HMEC-1 cells were cultured with endothelial cell medium from Procell Life Science & Technology Co. Ltd., (Wuhan, China). To investigate the effect of different glucose concentrations on the apoptosis of three kinds of cells, the cells were cultured with different concentrations of glucose-containing media for three days, and then stained with dUTP-FITC (fluorescein labeled deoxyuridine triphosphate) from a TUNEL kit (Beyotime, C1088) for green fluorescence imaging of apoptotic cells. To investigate the effect of VIS-photocatalytic therapy on the apoptosis in high-glucose conditions, cells were seeded in 24-well plates and treated with the high-glucose medium containing HTON, followed by VIS irradiation (0.1 W/cm^2^, 3 min). After 3 days of incubation, cells were stained with the TUNEL kit to determine the apoptosis.

### Measurement of the in vitro and in vivo levels of AGEs

The concentration of AGEs was measured according to the Lambert-Beer law based on a characteristic fluorescence at 470 nm (excited at 370 nm). AGEs (Abcam) solutions with different concentrations (6.25–100 μg/mL) were prepared and their fluorescence spectra were collected, followed by the building of the relationship between concentration and fluorescence intensity for the plot of the standard curve. Both the cells after 3 days of culture and collected skin tissues were lysed with the PBS buffer and centrifugated at 10,000 × *g* for 10 min at 4 °C. The supernatant was collected for fluorescence measurement in a fluorescence spectrophotometer (Thermo), and its AGEs concentration was calculated according to the above standard curve.

### Measurement of the RAGE level

HMEC-1 cells were seeded in a T25 plate and treated with the high-glucose medium containing HTON, followed by VIS irradiation (0.1 W/cm^2^, 3 min). After 3 days of incubation, cells were fully scraped off from the plate by cell curettage, lysed with PBS buffer solution, and then centrifuged at 4 °C at 15,000 × *g* for 10 min. The supernatant was collected and the RAGE concentration was determined by a RAGE Elisa Kit (Solarbio, SEKH-0302) following manufacturer’s protocol.

### mRNA sequencing measurement and analysis

HSF cells were cultured in a common incubator (74% N_2_, 21% O_2_ and 5% CO_2_) or a H_2_ incubator (60% H_2_, 14% N_2_, 21% O_2_ and 5% CO_2_), and then RNA was extracted by the TRIzol reagent. A mRNA library was constructed using a NEBNext Ultra RNA Library Prep Kit (Qiagen, Hilden, Germany) by following the protocol, and sequenced on an Illumina NovaSeq 6000 sequencer (CHI BIOTECH Co., Ltd). High-quality reads through Illumina filters were reserved for sequence analysis. Using Log2 (Fold Change) >1 and *P* < 0.05 as criteria, edgeR was used to screen differential mRNA. The heat map package of R language (V3.6.2) was used for cluster analysis of differentially-expressed mRNAs.

### qPCR measurement of the RAGE mRNA level

HSF cells were cultured in the common incubator or the H_2_ incubator, and then RNA was extracted by the TRIzol Reagent. The reverse transcription of RNA into complementary DNA (cDNA) was executed by using the First Strand cDNA kit (Takara) as protocol. The RAGE primers [5′ GGT CAT GCC GGA GTG TCT ATT 3′ (forward) and 5′ GTG TAG AAC CCG TCG GTG AGG 3′ (reverse)] were validated against and GAPDH primers [house keeping gene: 5′ CTG GAA AGC TGT GGC GTG ATG 3′ (forward) and 5′ GCC AGT GAG CTT CCC GTT CAG 3′ (reverse)] were used for PCR. Quantitative RT-PCR (qRT-PCR) was then performed using the Quantitect SYBR Green PCR master mix (Takara). The expression of RAGE gene was normalized to the GAPDH level, and the relative quantification of target mRNA was calculated by comparative cycle threshold (Ct).

### Measurement of cytotoxicity and cellular proliferation

To investigate the cytotoxicity of HTON to three kinds of cells, cells (1 × 10^4^ cells/well) were seeded in 96-well plates and incubated with culture media containing different concentrations of HTON (12.5–200 μg/mL) for 24 h. The CCK-8 reagent (Beyotime, Shanghai, China) was added into each well followed by 60-min incubation at 37 °C, and then the absorbance at 450 nm was collected on a BioTek Synergy HIM microplate reader (CA, USA). Cytotoxicity was expressed as the percentage of cell viability compared to blank control. Similarly, the cytotoxicity of STT and AA at the concentration of 31.2–1000 μg/mL was also measured. As to the cytotoxicity of the HTON@Gel dressing, the 20 μL freshly prepared dressing was paved at the bottom of a 96-well plate, and then incubated in the cell incubator for 10 min for natural gelling followed by 24-h cell culture (10^4^ cells/well) and CCK-8 test.

For cellular proliferation, 2 × 10^3^ cells per well were seeded in 96-well plates and incubated with high-glucose culture medium containing 200 μg/mL HTON for 7 days. In the HTON + VIS + STT and HTON + VIS + AA groups, 250 μg/mL STT and AA were individually added with HTON solution. For VIS-involved groups, cells were irradiated under the Xe lamp (0.1 W/cm^2^) for 3 min every day and CCK-8 was used to detect cell viability at different time points (0, 1, 3, 5, and 7 days).

### Measurement of cellular migration

In vitro cellular migration was evaluated by the scratch test. Cells were seeded in a 6-well plate, and immediately made a straight scratch by using a pipette tip to simulate a wound before overgrowth. Then cells were treated with the medium containing HTON followed by VIS irradiation (0.1 W/cm^2^, 3 min). After incubation for 6–36 h, the migration distance of cells was observed under an inverted microscope (Nikon, FHEIPSE, Japan), and the relative migration area was calculated compared with blank control by Image J software.

### Diabetic mouse model building and treatment

The protocol was approved by Animal Ethical and Welfare Committee of Shenzhen University (No. 2021005). C57BL/6 male mice (4 weeks old) were purchased from the Model Animal Research Center of Guangdong University and housed in groups (four to six) in plastic cages on a 12-h light/dark cycle. The temperature and humidity were kept at 24–26 °C and 60%, respectively. After 1-week gradual addition of high-fat diet, mice were feed with high-fat diet continuously for 1 month under a specific pathogen-free environment. STZ at a dose of 50 mg/kg/day was injected intraperitoneally into the mice for 5 days to induce diabetes. After 10 days, the blood glucose levels were measured by using a glucose meter. The mice were considered diabetic when the fasted plasma glucose levels were stably higher than 20 mM.

The mice were anesthetized in a chamber (RWD Life Science Co., LTD, China) containing 1.5% isoflurane and 60% oxygen. Excisional dorsal skin wounds were made with a 10 mm sterile biopsy punch and then fixed with a circular rubber ring to prevent the skin from retraction, followed by treatment. The diabetic model mice were assigned randomly into five groups (*n* = 4): diabetic control, Gel, Gel + VIS (0.05 W/cm^2^, 15 min VIS irradiation every other day), HTON@Gel, and HTON@Gel + VIS (0.05 W/cm^2^, 15 min VIS irradiation every other day). In addition, a normal wound without diabetes was used as a normal control group. Each mouse was kept in an individual cage with plentiful water and food, and observed daily during the whole period of treatment. After treatment for 7 and 14 days, mice were euthanized and their skins at the wound site were collected and then fixed in 4% paraformaldehyde followed by paraffin embedding. Representative skin sections were stained with hematoxylin & eosin (H&E) and Masson’s trichrome following routine protocols, and then observed on an optical microscope (Olympus BX-41/Q-Color3, Japan).

### Western blot analysis

The collected skin tissues were lysed using the RIPA lysis buffer containing phenylmethanesulfonyl fluoride (PMSF), and then centrifugated at 10,000 × *g* for 15 min at 4 °C. The supernatant was collected for measurement of the protein concentration with a BCA protein assay kit, followed by Western blot analysis. The equal amount of protein extract (15 μg/well) in each sample was separated with SDS-PAGE and then transferred to the polyvinylidene fluoride (PVDF) membrane. After blocked with 5% skim milk for 30 min, the protein was incubated with the corresponding primary antibody overnight at 4 °C and then with the enzyme-conjugated secondary antibody for 1 h. Blots were demonstrated by the ECL reagents, and then visualized on an Image Station (Tanon-5200, China). Image J software was used to quantify the intensity of bands.

### Immunofluorescence staining assay

Paraffin-embedded skin tissues were sectioned into pieces and then mounted on slides. After permeabilized with the immunostaining permeable fluid (Beyotime, P0097) for 15 min, slides were blocked with the PBST buffer containing 2% BSA for 15 min, and incubated with the corresponding primary antibody overnight at 4 °C and then with the second antibody for 1 h. Finally, slides are sealed with a drop of sealing tablet and imaged on a confocal laser scanning microscope (LEICA-SP5II).

### Biochemical measurement

Blood samples were centrifuged at 3000 × *g* at 4 °C for 15 min. Cleared plasma was aliquoted and stored at −80 °C for use. Liver and kidney functions were measured on a biochemical analyzer (iMagic-M7). Aftar anticoagulant was added into the whole blood, hematology markers were measured on a blood cell analyzer (BC-31s, Mindray)

### Statistical analysis

Statistical analysis was performed using GraphPad Prism 9.0 software (GraphPad Inc., San Diego, CA, USA). Quantitative data were analyzed using the one-way ANOVA test. *P* < 0.05 was considered to be a statistically significant difference. Experimental data were obtained from at least three independent replicates.

### Reporting summary

Further information on research design is available in the Nature Research Reporting Summary linked to this article.

## Supplementary information

Supplementary Information

Reporting Summary

## Data Availability

All the data supporting the findings of this study are available within the article and its Supplementary Information files and from the corresponding author upon reasonable request. A reporting summary for this article is available as a Supplementary Information file. The uncropped and unprocessed western blot scans for Supplementary Fig. 31a are provided in the Source Data file. Source Data are provided with this paper.

## Source data

Source Data
