# Supplementary material for: Photocatalytic glucose depletion and hydrogen generation for diabetic wound healing
Source: Nat Commun. 2022 Sep 27;13:5684. doi: 10.1038/s41467-022-33475-7 (PMC9515190; doi:10.1038/s41467-022-33475-7)
Supplement: Supplementary file 1 — Supplementary Information [file 41467_2022_33475_MOESM1_ESM.pdf]

Supplementary Information

Photocatalytic glucose depletion and hydrogen generation for diabetic wound healing

Shengqiang Chen,[1,†] Yanxia Zhu,[2,†] Qingqing Xu,[1] Qi Jiang,[1] Danyang Chen,[3] Ting Chen,[1] Xishen Xu,[1] Zhaokui Jin,[1] Qianjun He[1,3,4*]

[1] Guangdong Key Laboratory for Biomedical Measurements and Ultrasound Imaging, School of Biomedical Engineering, Health Science Center, Shenzhen University, Shenzhen 518060, China

[2] Department of Cell Biology and Medical Genetics, School of Basic Medical Sciences, Shenzhen University Health Science Center, Shenzhen, 518060, China

[3] Center of Hydrogen Science, School of Materials Science and Engineering, Shanghai Jiao Tong University, Shanghai 200240, China

[4] Shenzhen Research Institute, Shanghai Jiao Tong University, Shenzhen 518057, China

[†] These authors contributed equally to this work.

* Corresponding author. Email: nanoflower@126.com (Qianjun He)

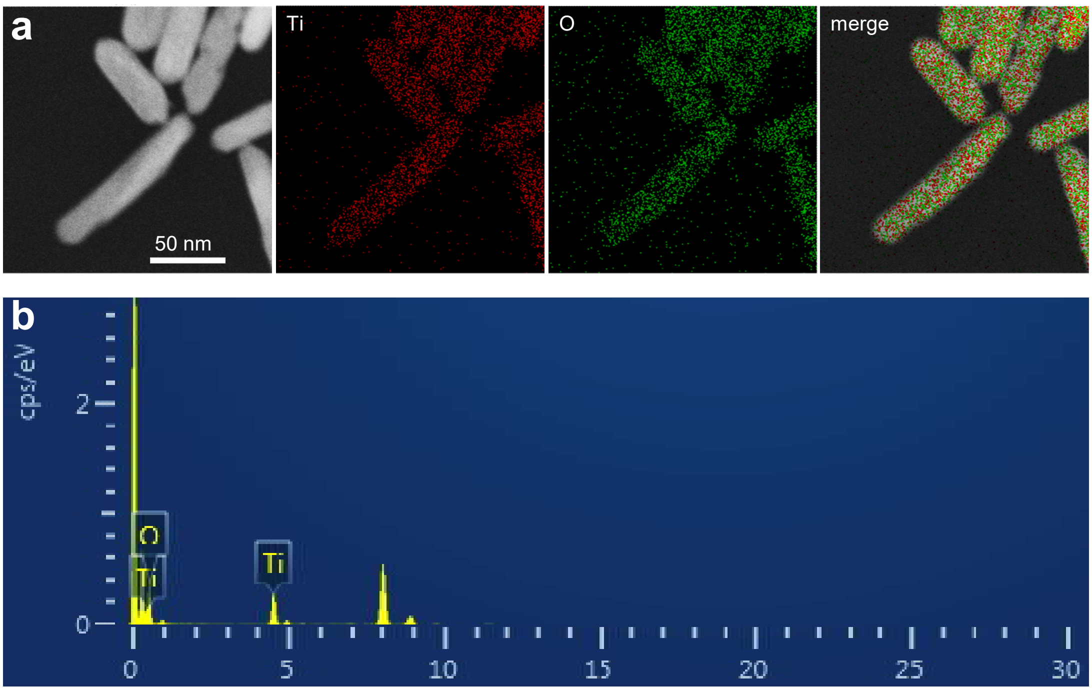

Supplementary Figure 1. HADDF and corresponding elementary mapping images of HTON. The experiments were repeated three times independently with similar results.

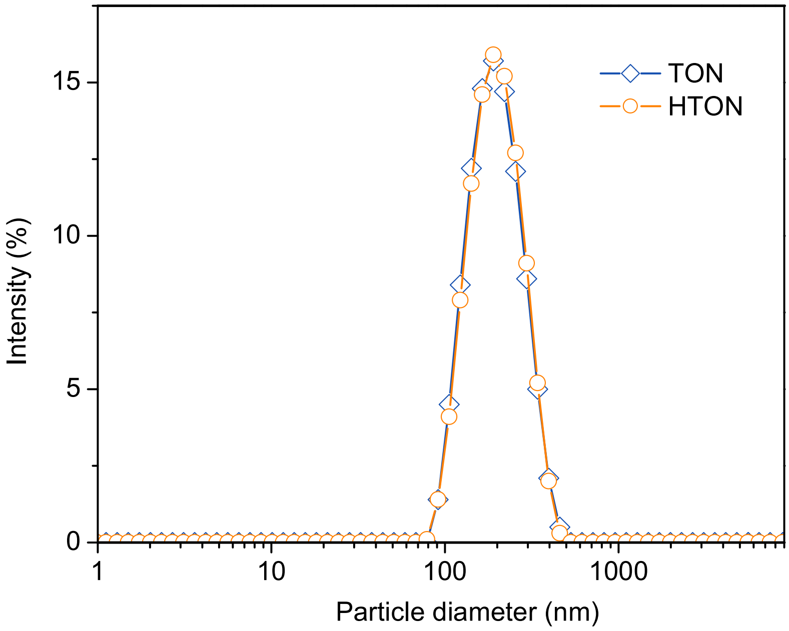

Supplementary Figure 2. The particle size distribution of TON and HTON by dynamic light scattering.

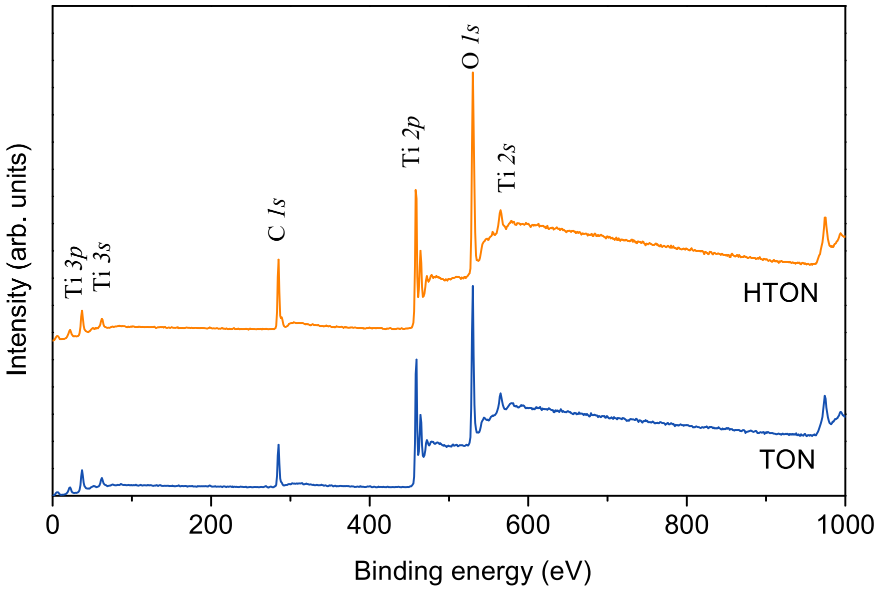

Supplementary Figure 3. XPS patterns of TON and HTON.

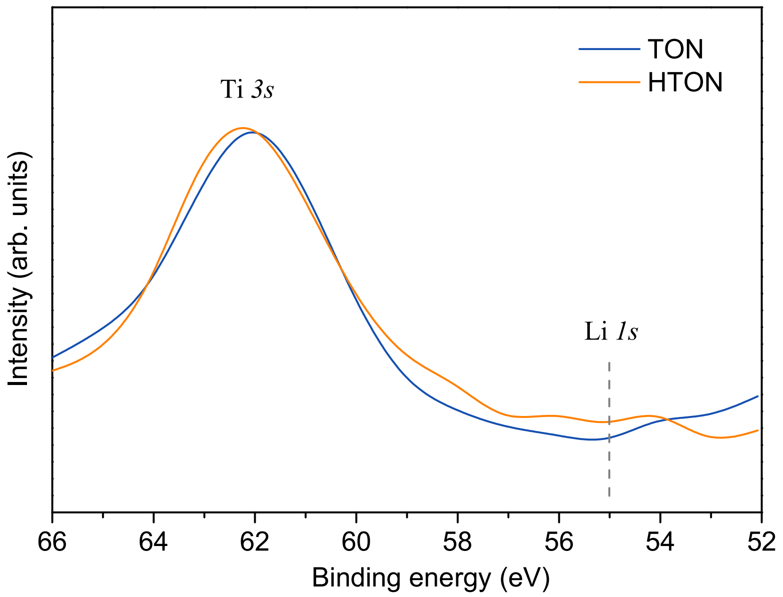

Supplementary Figure 4. XPS spectra of TON and HTON for identification of Li.

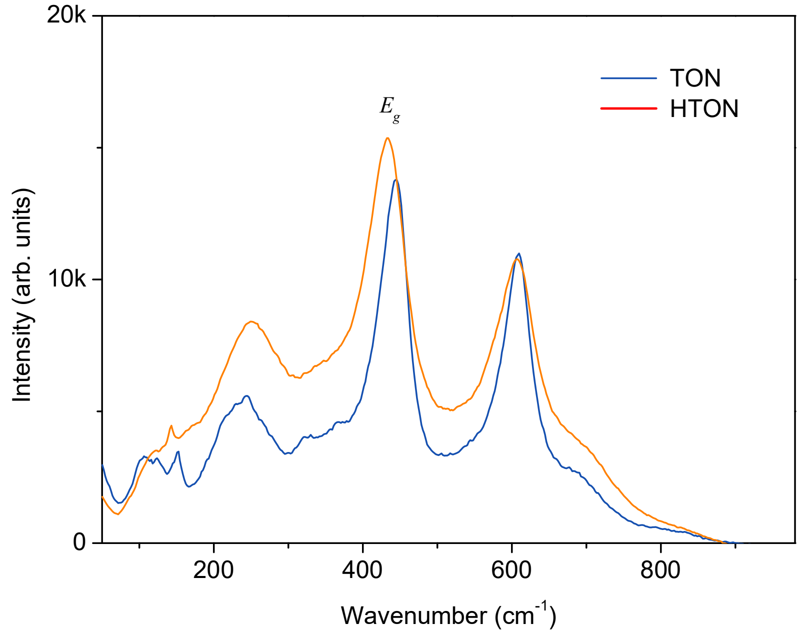

Supplementary Figure 5. Raman spectra of TON and HTON.

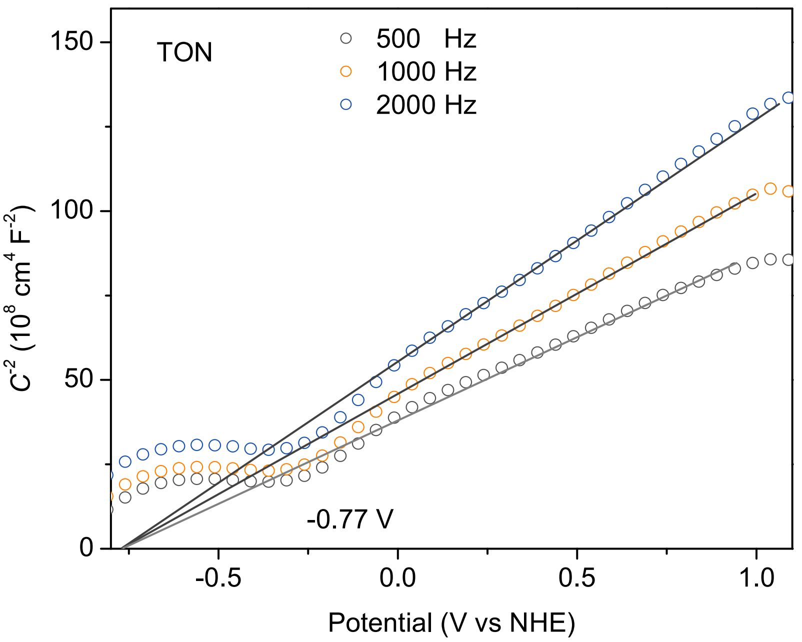

Supplementary Figure 6. Mott–Schottky plots of TON (vs NHE).

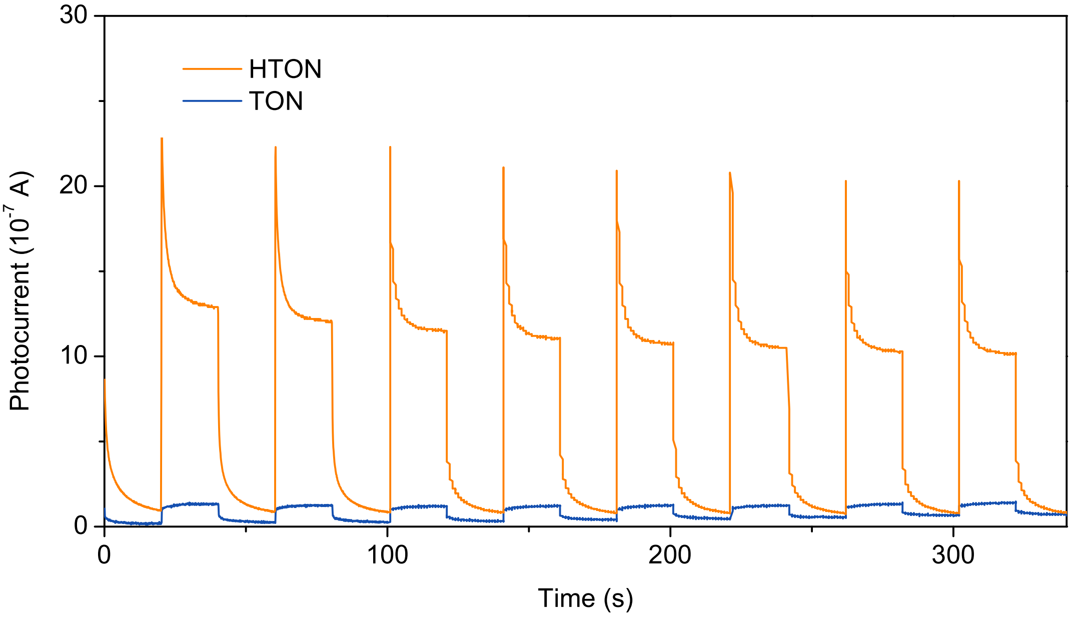

Supplementary Figure 7. Transient photocurrent (I–T) curves of TON and HTON.

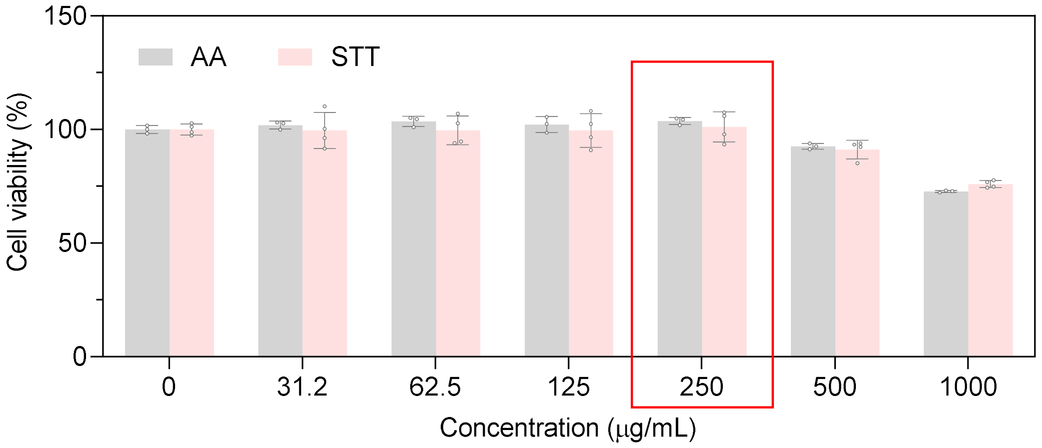

Supplementary Figure 8. The cytotoxicity of AA (*L*-ascorbic acid) ($n = 3$ biologically independent samples) and STT (sodium tetrathionate) ($n = 4$ biologically independent samples) against HMEC-1 cells. Data were presented as mean value ± SD.

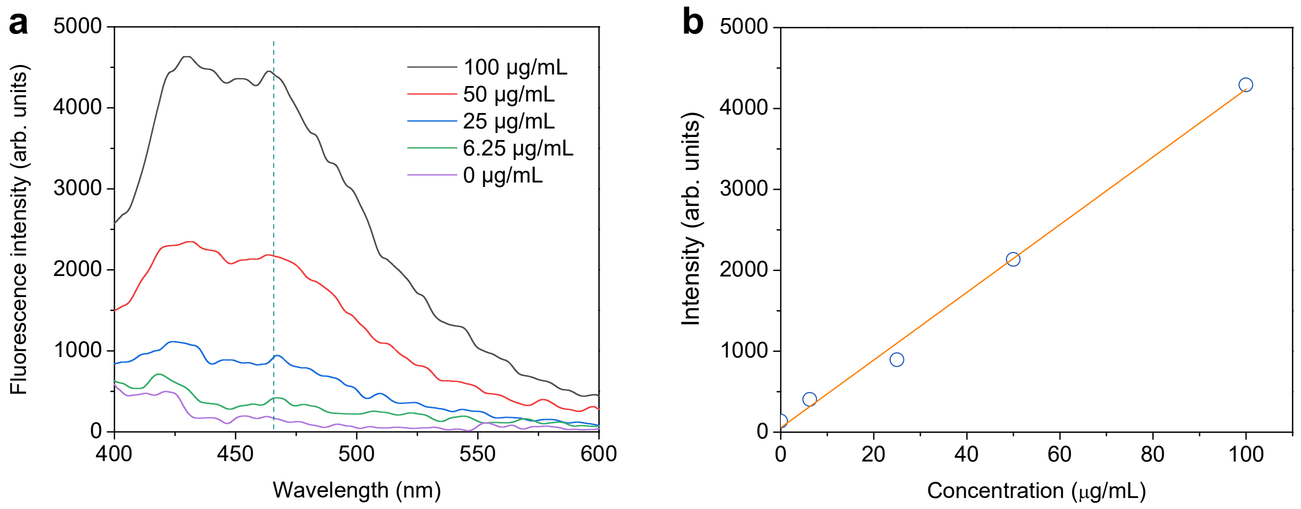

Supplementary Figure 9. The standard curve for AGEs.

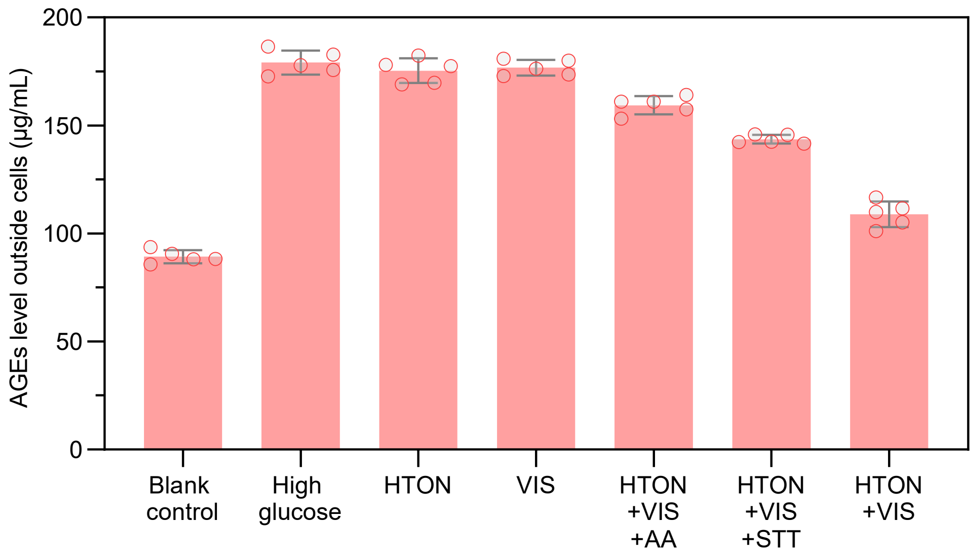

Supplementary Figure 10. The AGEs levels outside treated cells. *L*-ascorbic acid (AA) and sodium tetrathionate (STT) were used as hole-sacrificial and electron-sacrificial agents, respectively ($n = 5$ biologically independent samples). Data were presented as mean value ± SD.

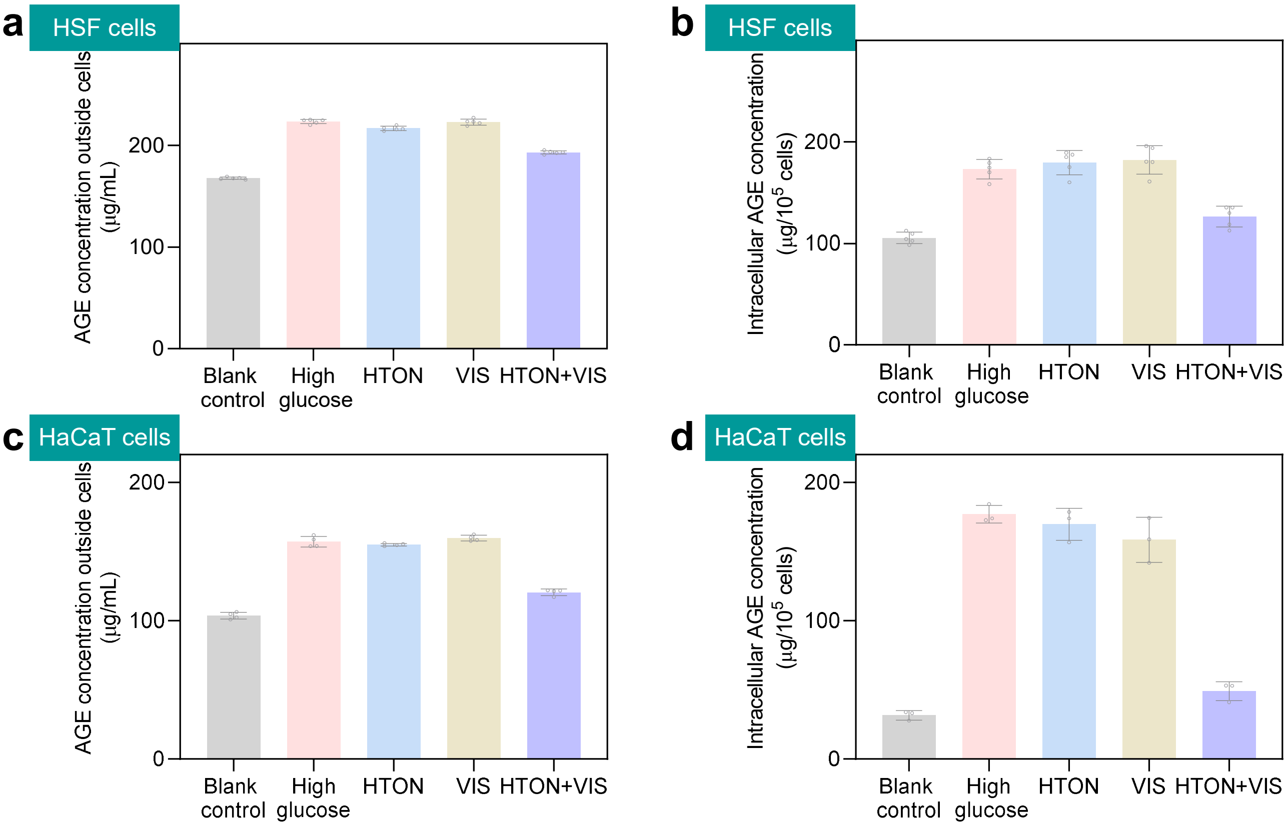

Supplementary Figure 11. The AGEs concentrations inside (**b**,**d**) and outside (**a**,**c**) HSF (**a**,**b**) and HaCaT cells (**c**,**d**) after treatment (n=5 biologically independent samples for figure a, n=5 biologically independent samples for figure b, n=4 biologically independent samples for figure c, and n=3 biologically independent samples for figure d). Data were presented as mean value ± SD.

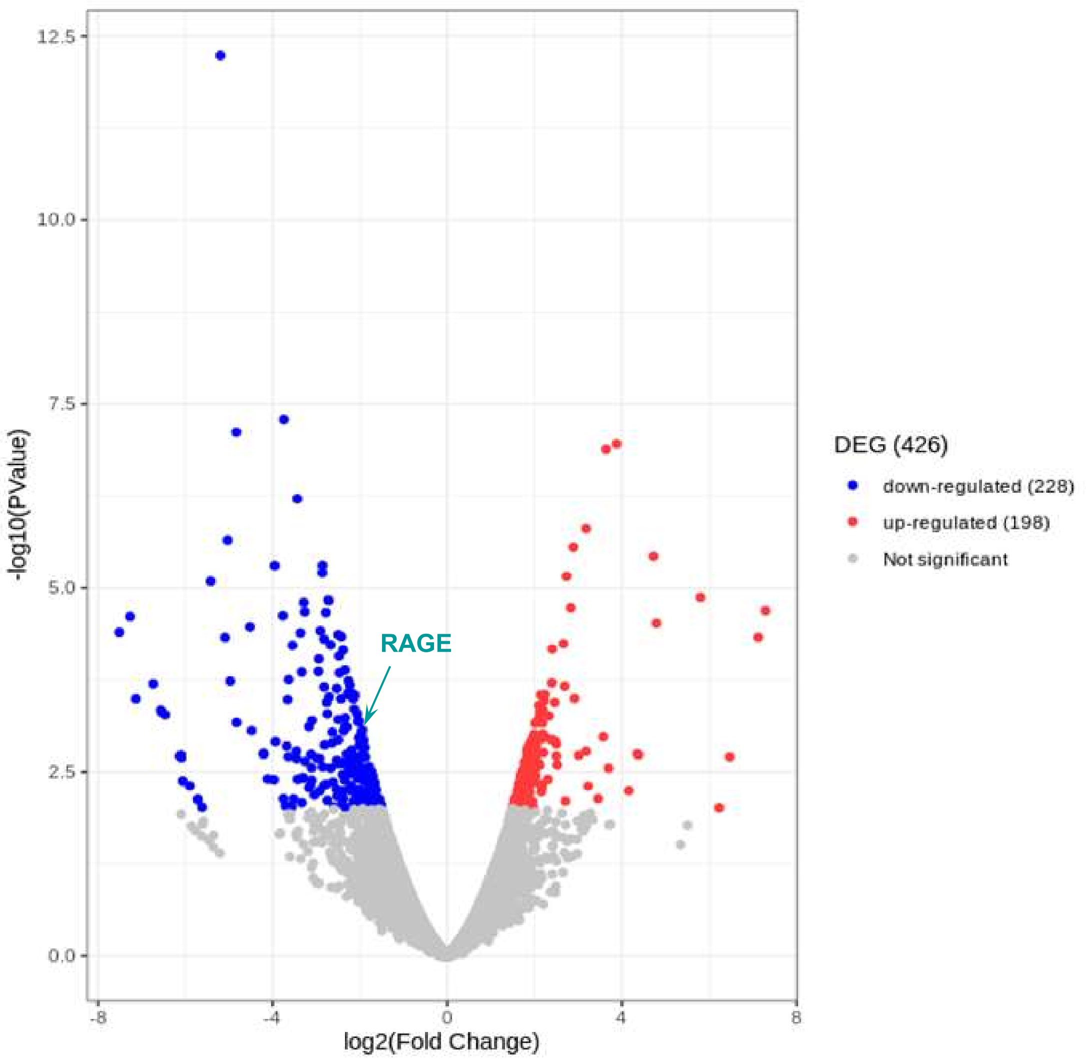

Supplementary Figure 12. The Volcano map of differentially expressed genes by using the RNAseq technique to screen differentially-expressed genes in HSF cells before and after treatment with hydrogen molecules. The input data of differential expression analysis are ReadCount data obtained from gene expression level analysis. We used edgeR for analysis, and the analysis method was based on negative binomial distribution. In order to eliminate biological variation, the screening of differential genes should be evaluated in terms of difference multiple and significant level. In this analysis, the screening threshold of differential genes was set to $p < 0.01$ and $|\log2\ (\text{FoldChange})| > 1$.

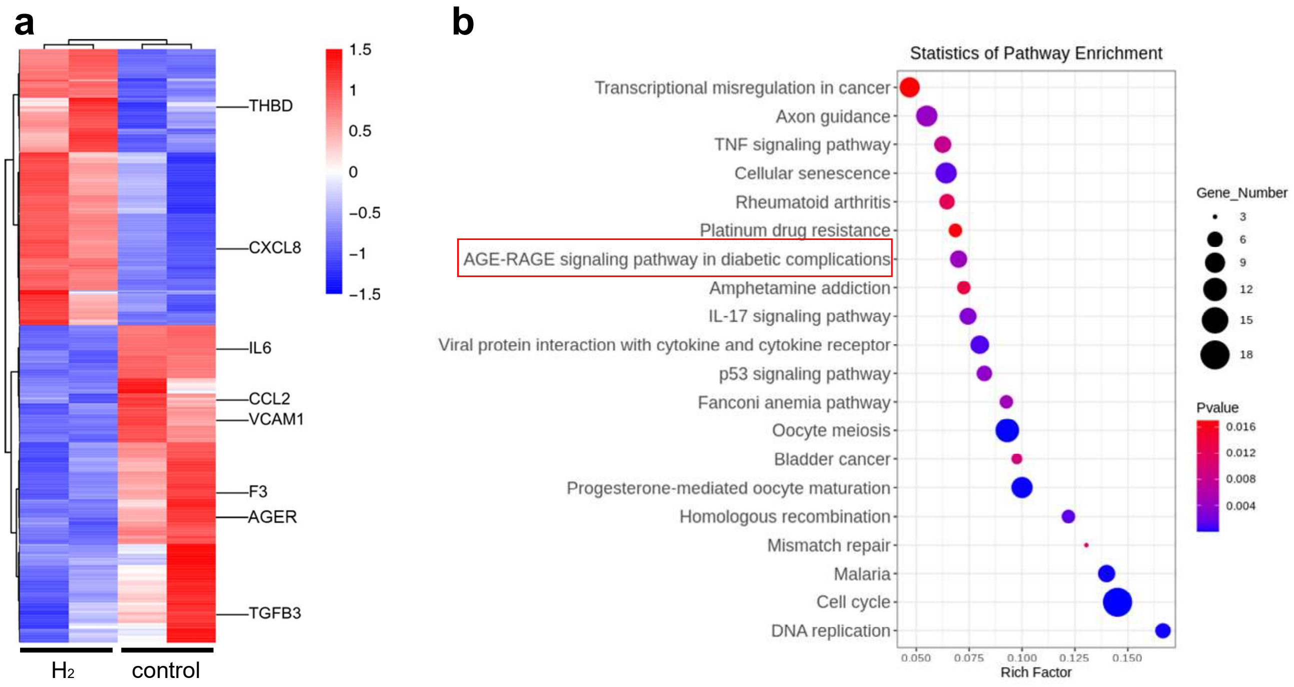

Supplementary Figure 13. The cluster diagram of differential genes (**a**), and rich distribution map of KEGG genes (**b**) after treatment with hydrogen molecules. We carried on the KEGG pathway enrichment analysis to screen differential genes, the analysis software was clusterProfiler, and the statistical test method was Hypergeometric Test. The Rich Factor in the picture refers to the ratio of the number of differential genes enriched in the pathway to the number of annotated genes. The larger the Rich Factor, the greater the degree of enrichment.

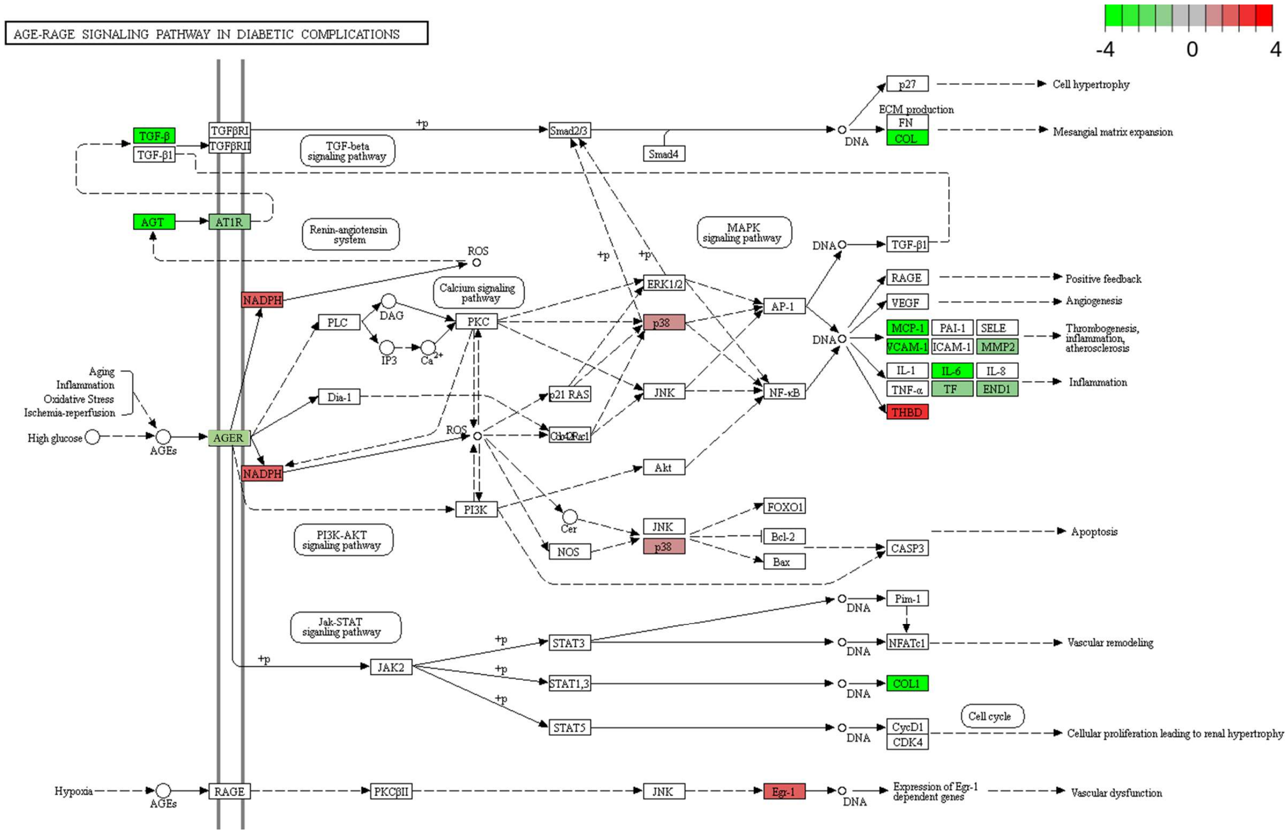

Supplementary Figure 14. The AGE-RAGE signaling pathway in diabetic complications after treatment with hydrogen molecules.

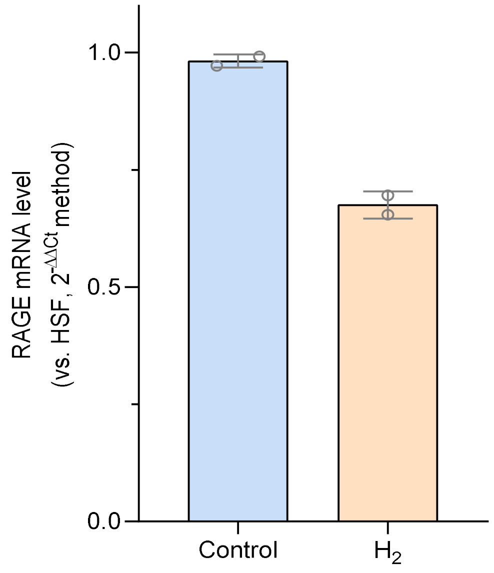

Supplementary Figure 15. The RAGE mRNA level in HSF cells before and after treatment with hydrogen molecules ($n = 2$ biologically independent samples). Data were presented as mean value ± SD.

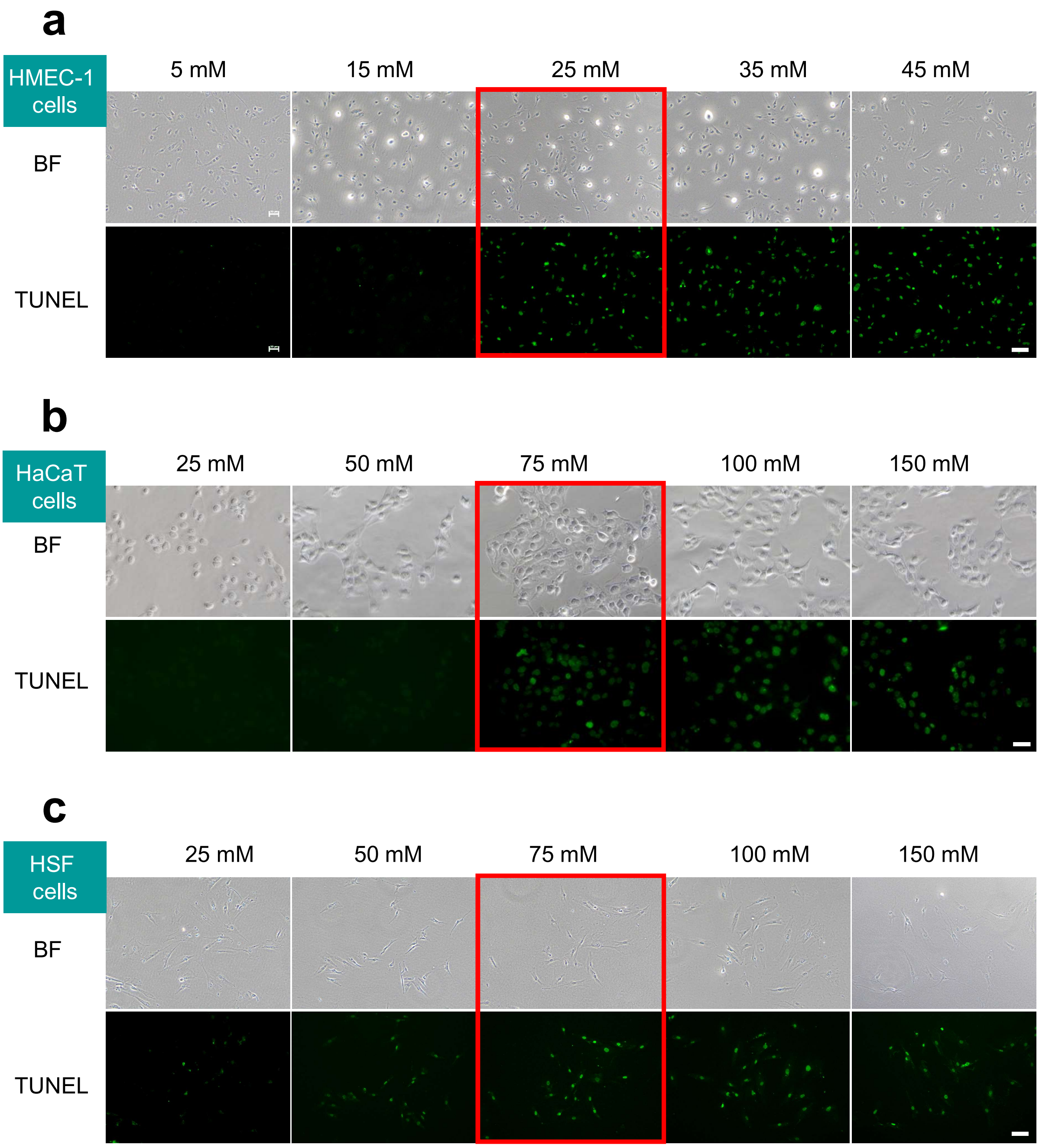

Supplementary Figure 16. The high glucose-induced apoptosis of HMEC-1 (**a**), HaCaT (**b**), and HSF (**c**) cells. Scale bars, 100 µm. The experiments for figures a-c were repeated three times independently with similar results.

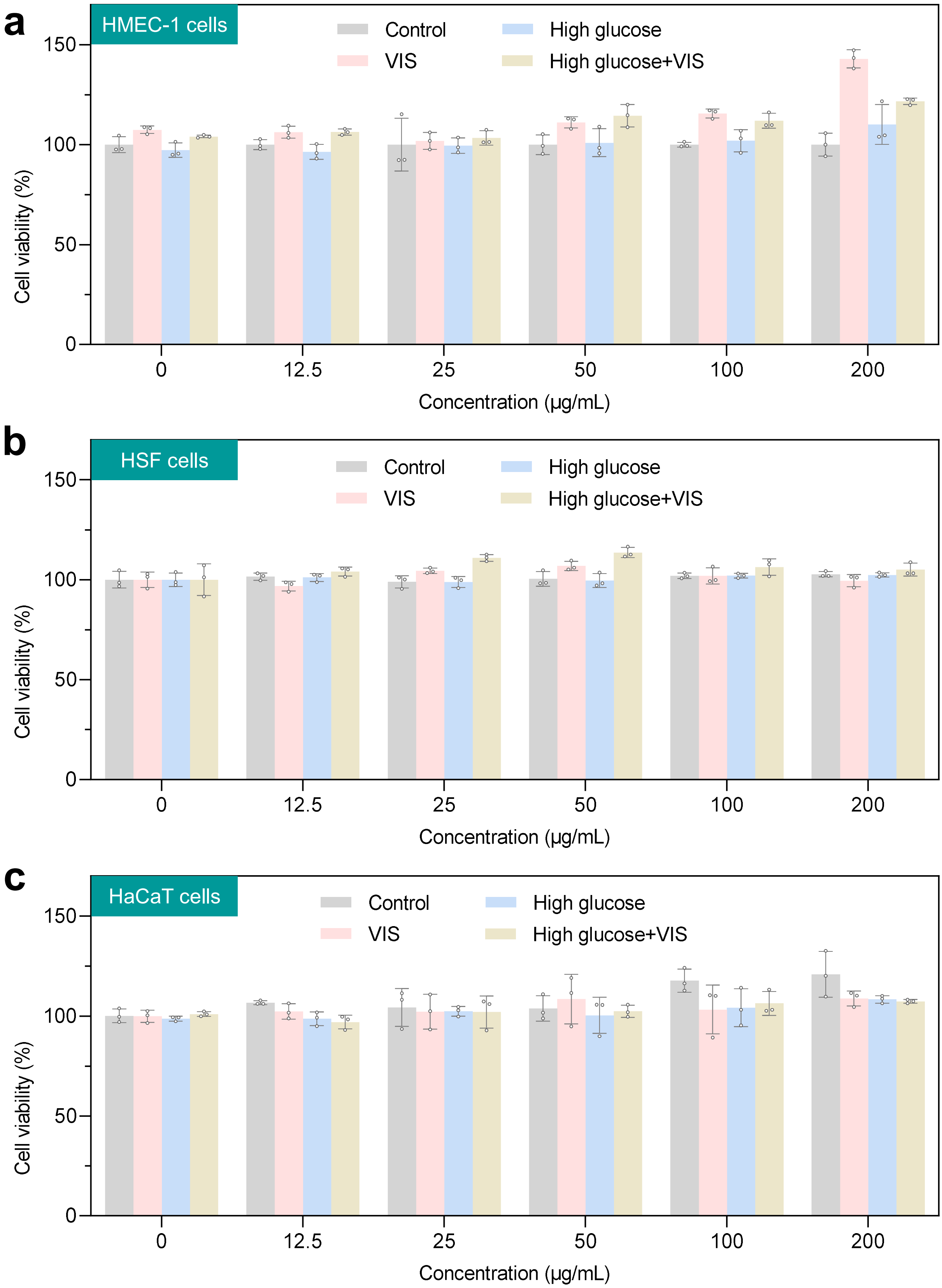

Supplementary Figure 17. The cytotoxicity of HTON to HMEC-1 (**a**), HSF (**b**), and HaCaT (**c**) cells ($n = 3$ biologically independent samples). Data were presented as mean value ± SD.

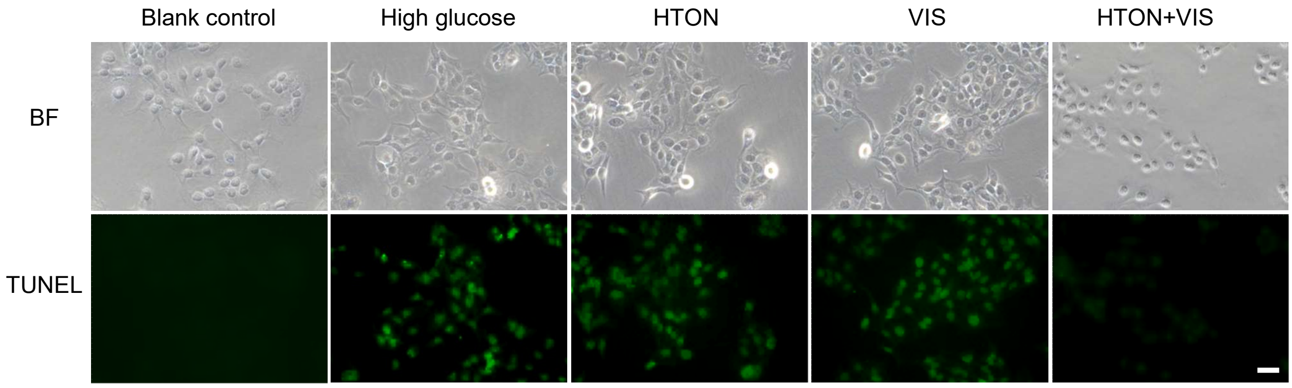

Supplementary Figure 18. The effect of VIS-photocatalytic therapy on the apoptosis of HaCaT cells. Scale bar, 100 μm. The experiments were repeated three times independently with similar results.

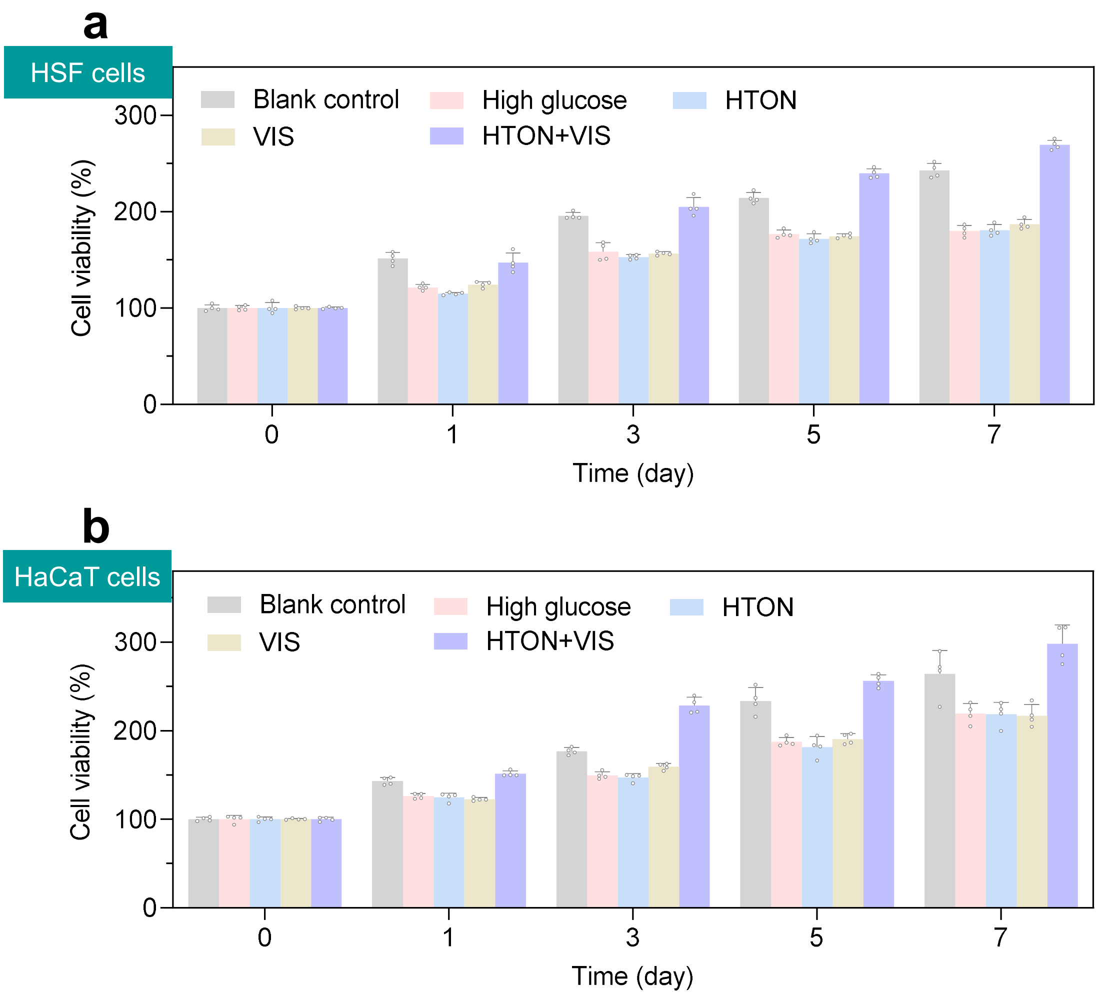

Supplementary Figure 19. The effect of VIS-photocatalytic therapy on the proliferation of HSF (**a**) and HaCaT (**b**) cells ($n = 4$ biologically independent samples). Data were presented as mean value ± SD.

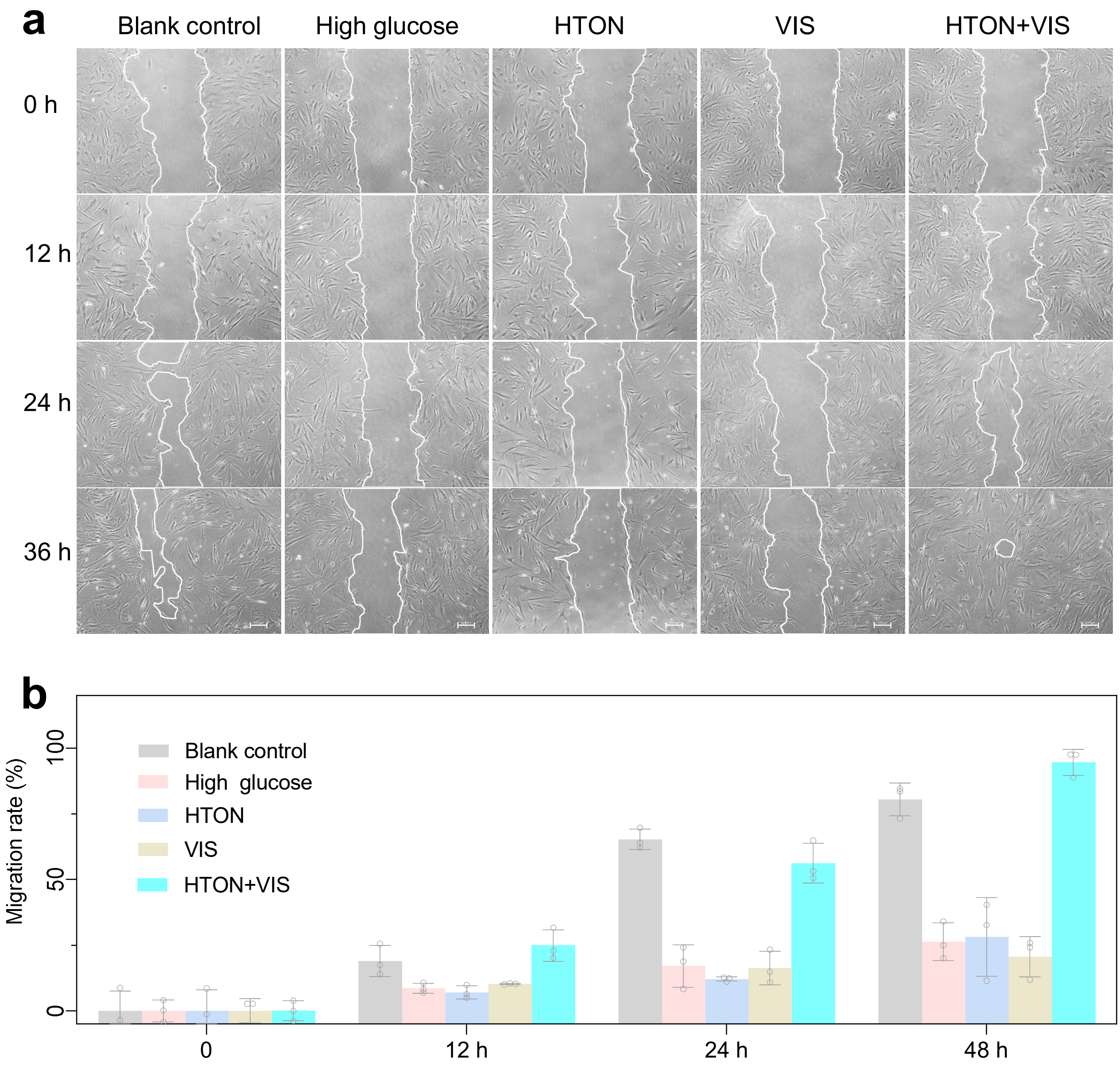

Supplementary Figure 20. The effect of VIS-photocatalytic therapy on the migration of HSF cells (**a**), and corresponding statistical analysis ($n = 3$ biologically independent samples) (**b**). Scale bar, 200 μm. Data were presented as mean value ± SD.

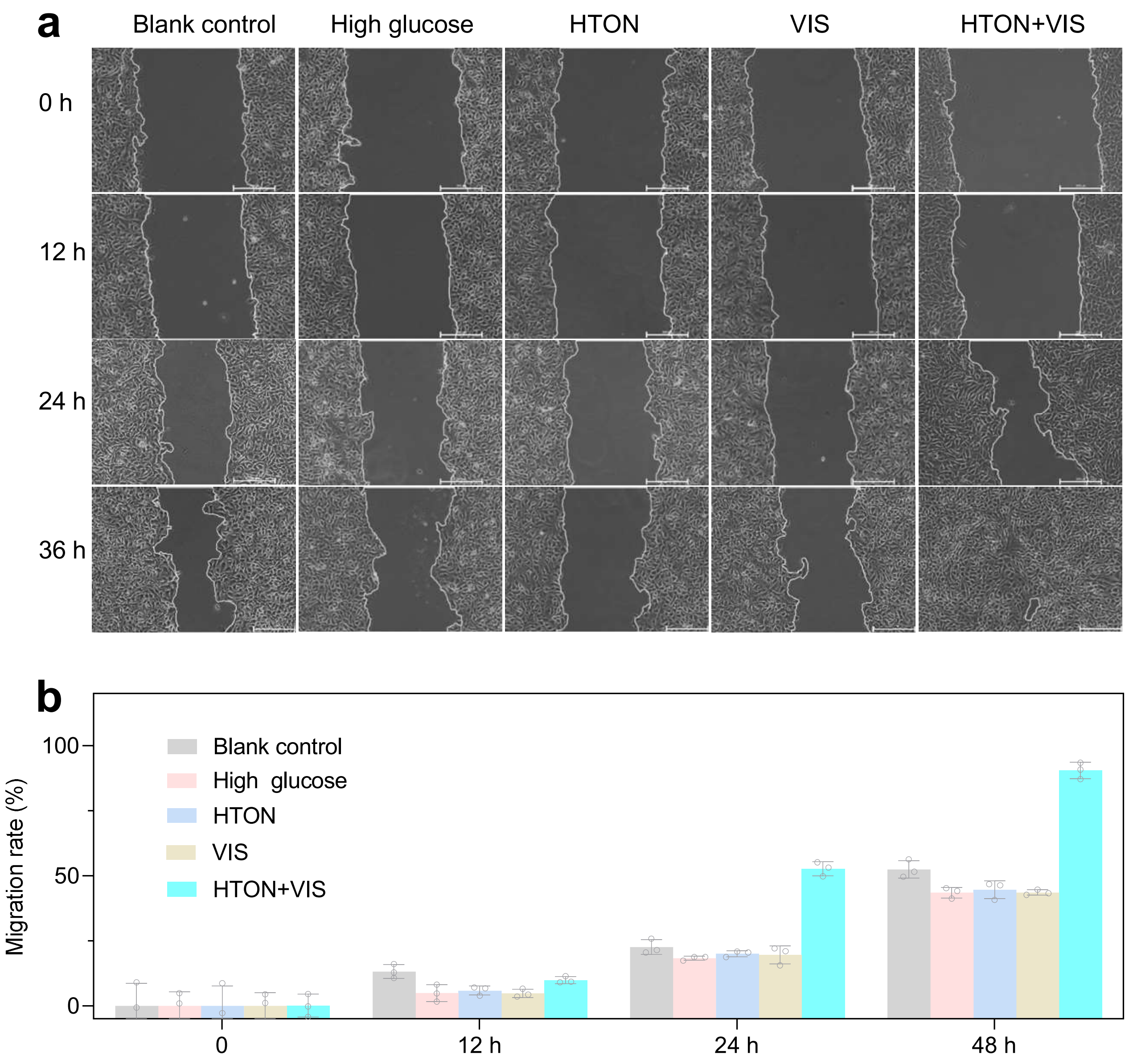

Supplementary Figure 21. The effect of VIS-photocatalytic therapy on the migration of HaCaT cells (**a**), and corresponding statistical analysis ($n = 3$ biologically independent samples) (**b**). Scale bar, 200 μm. Data were presented as mean value ± SD.

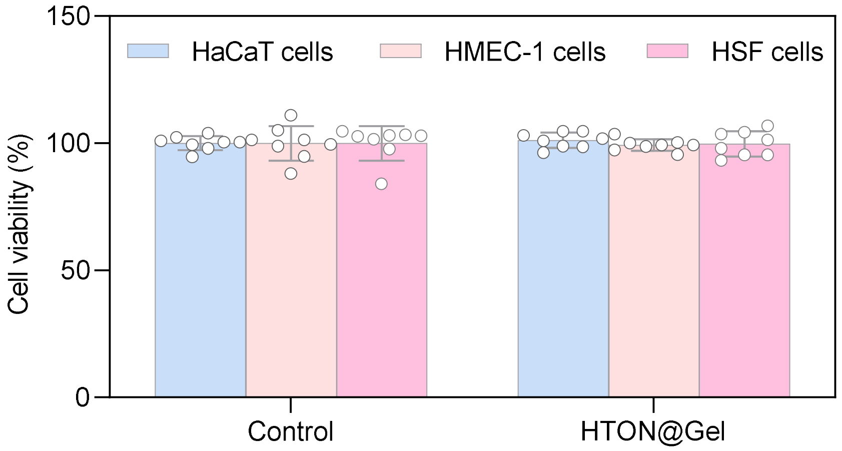

Supplementary Figure 22. The viability of HMEC, HSF and HaCaT cells after incubation with the HTON@Gel dressing for 24 h ($n = 8$ biologically independent samples). Data were presented as mean value ± SD.

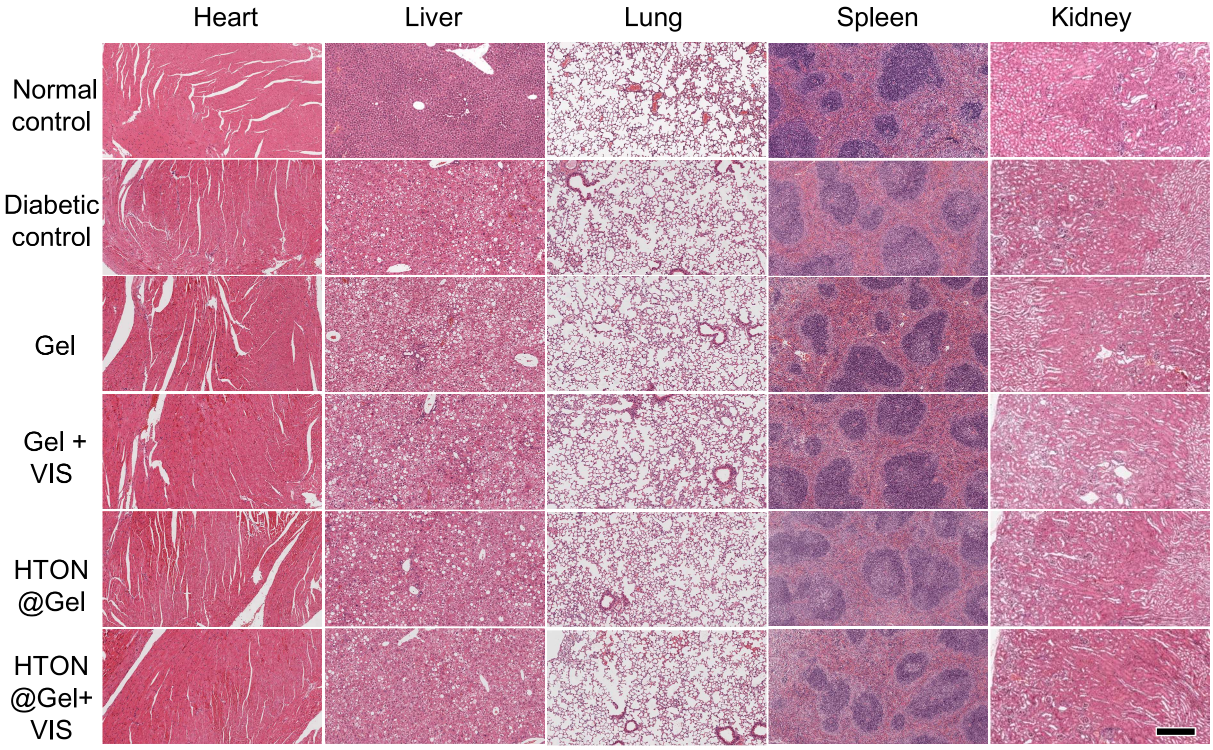

Supplementary Figure 23. HE staining images of main organs (heart, lung, liver, kidney and spleen) after 14-day treatment. Scale bar, 500 μm. The experiments were repeated three times independently with similar results.

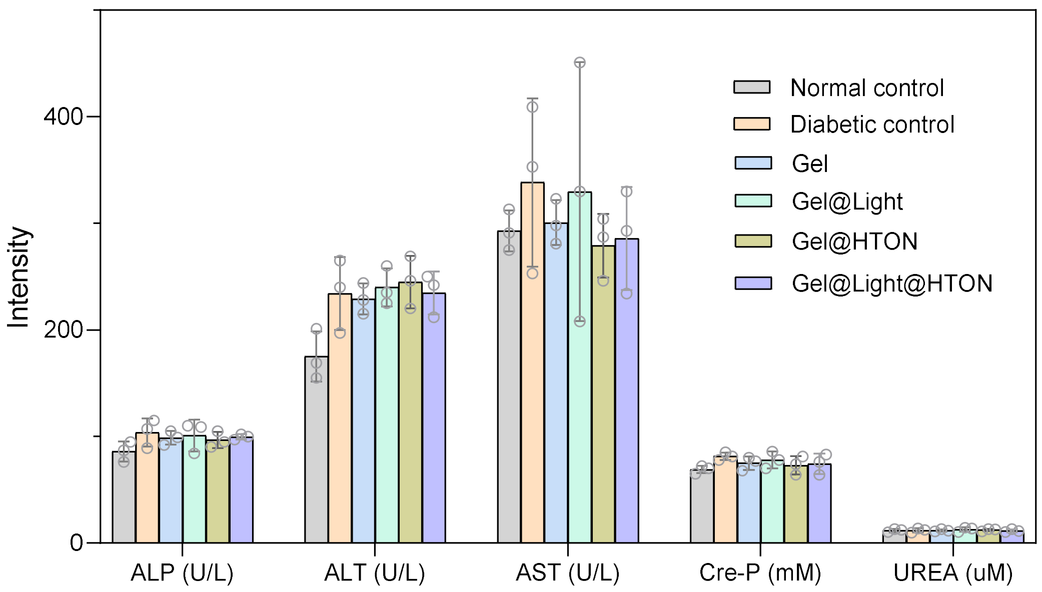

Supplementary Figure 24. Blood biochemical analyses of liver/kidney functions. ALP, alkaline phosphatase; ALT, alanine transaminase; AST, aspartate transaminase; Cre-P, creatinine; UREA, blood urea ($n = 3$ biologically independent samples). Data were presented as mean value ± SD.

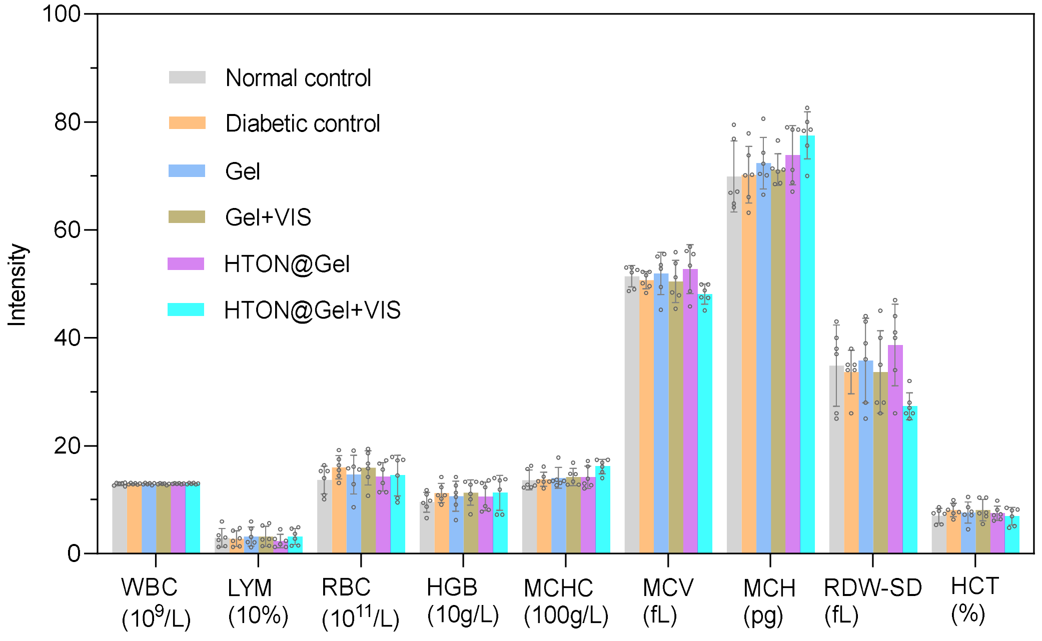

Supplementary Figure 25. The assessment of major hematology markers including white blood cells (WBC), red blood cells (RBC), hemoglobin (HGB), hematocrit (HCT), mean corpuscular volume (MCV), mean corpuscular hemoglobin (MCH), red blood cell volume distribution width (RDW-SD) and lymphocytes percentage (LYM) ($n = 6$ biologically independent samples). Data were presented as mean value ± SD.

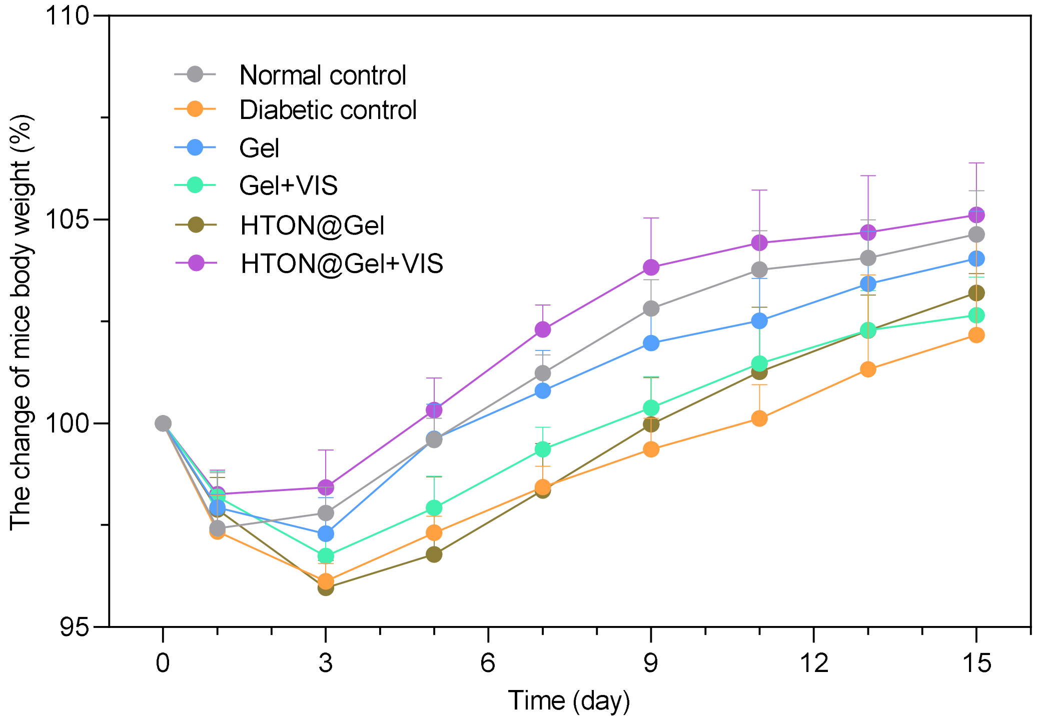

Supplementary Figure 26. The body weight change of different treatment groups during treatment ($n = 5$ biologically independent samples). Data were presented as mean value ± SD.

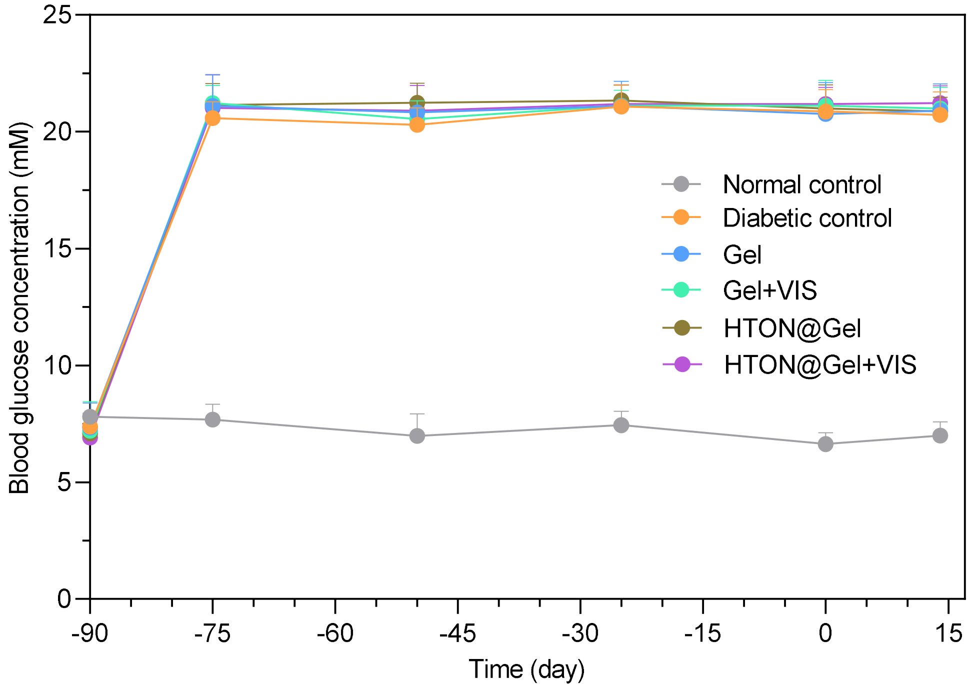

Supplementary Figure 27. The blood glucose concentration change during model building and the treatment ($n = 5$ biologically independent samples). Data were presented as mean value ± SD.

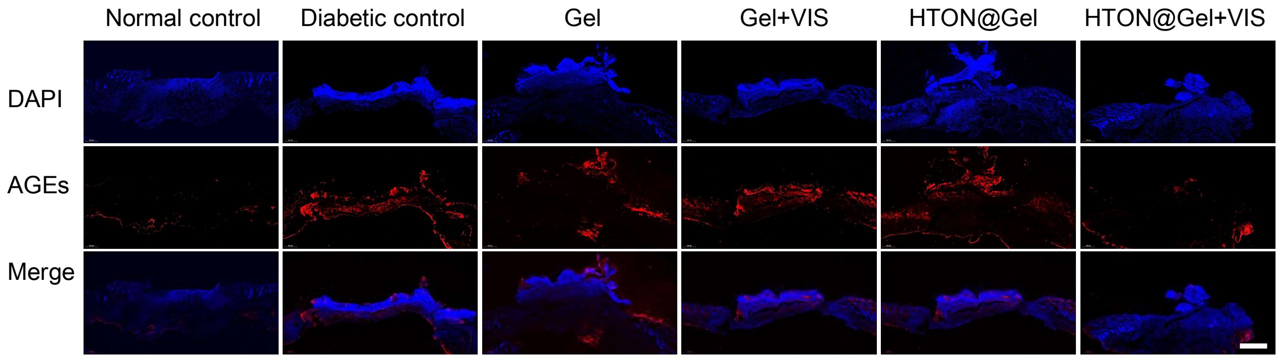

Supplementary Figure 28. The AGEs level at the diabetic wound site at Day 7 indicated by immunofluorescence staining. Scale bar, 1000 μm. The experiments were repeated three times independently with similar results.

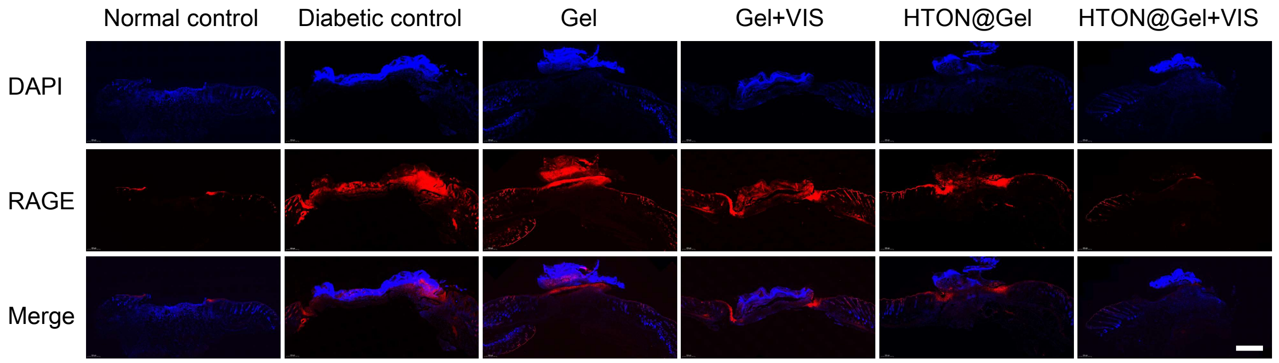

Supplementary Figure 29. The RAGE level at the diabetic wound site at Day7 indicated by immunofluorescence staining. Scale bar, 1000 μm. The experiments were repeated three times independently with similar results.

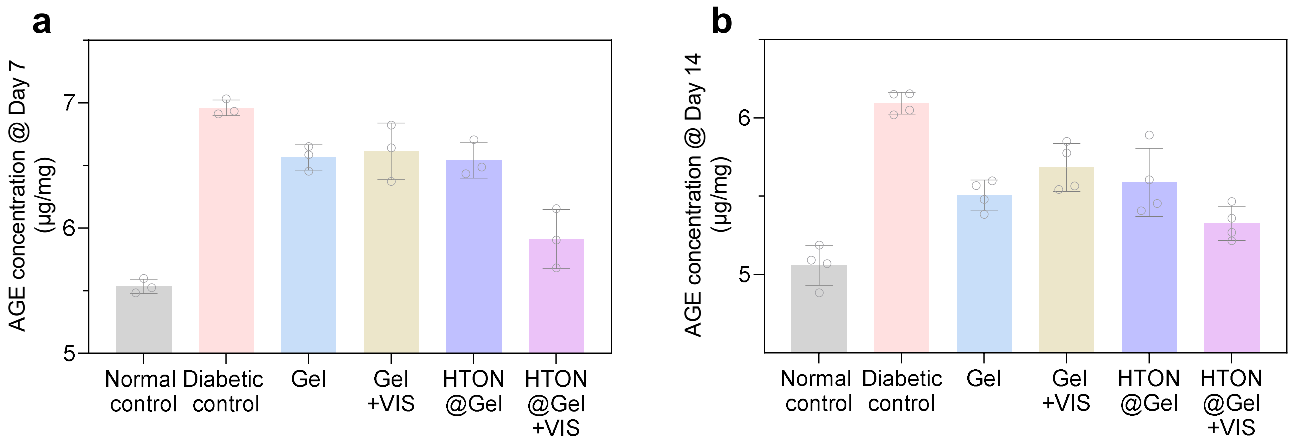

Supplementary Figure 30. The AGEs level in diabetic wound tissue at Day 7 ($n = 3$ biologically independent samples) (**a**) and Day 14 ($n = 4$ biologically independent samples) (**b**). Data were presented as mean value ± SD.

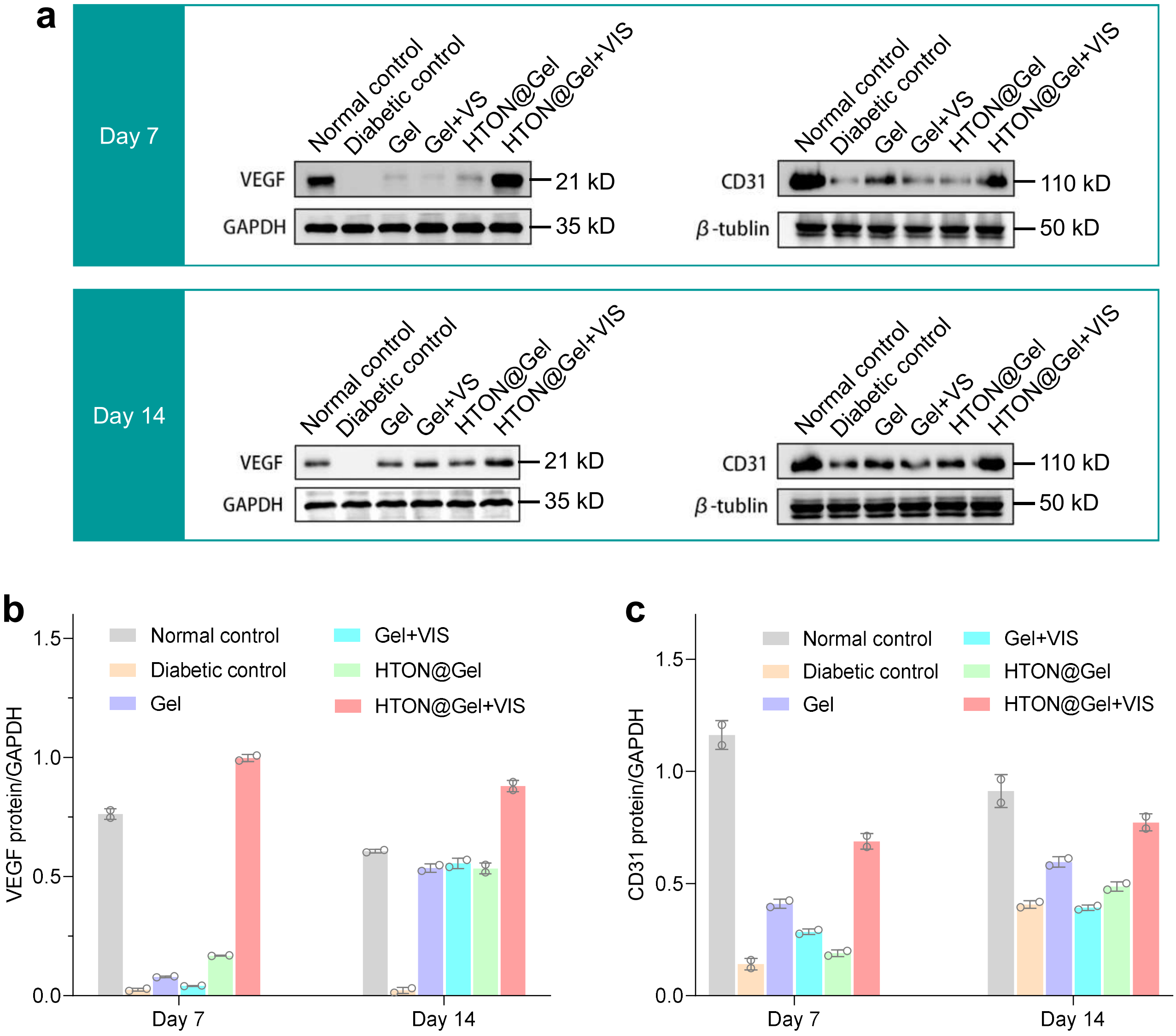

Supplementary Figure 31. The VEGF and CD31 levels at the diabetic wound site at Day 7 and Day 14 indicated by Western blot (**a**), and corresponding statistical analysis (**b**,**c**) ($n = 2$ biologically independent samples). Data were presented as mean value ± SD.
