# Supplementary material for: Photocatalytic glucose depletion and hydrogen generation for diabetic wound healing
Source: Nat Commun. 2022 Sep 27;13:5684. doi: 10.1038/s41467-022-33475-7 (PMC9515190; doi:10.1038/s41467-022-33475-7)
Supplement: Supplementary file 2 — Reporting Summary [file 41467_2022_33475_MOESM2_ESM.pdf]

# nature portfolio

Corresponding author(s): Qianjun He

Last updated by author(s): Aug 30, 2022

# Reporting Summary

Nature Portfolio wishes to improve the reproducibility of the work that we publish. This form provides structure for consistency and transparency in reporting. For further information on Nature Portfolio policies, see our Editorial Policies and the Editorial Policy Checklist.

## Statistics

For all statistical analyses, confirm that the following items are present in the figure legend, table legend, main text, or Methods section.

| n/a | Confirmed | |
|---|---|---|
| ☐ | ☒ | The exact sample size ($n$) for each experimental group/condition, given as a discrete number and unit of measurement |
| ☐ | ☒ | A statement on whether measurements were taken from distinct samples or whether the same sample was measured repeatedly |
| ☐ | ☒ | The statistical test(s) used AND whether they are one- or two-sided<br>*Only common tests should be described solely by name; describe more complex techniques in the Methods section.* |
| ☒ | ☐ | A description of all covariates tested |
| ☒ | ☐ | A description of any assumptions or corrections, such as tests of normality and adjustment for multiple comparisons |
| ☐ | ☒ | A full description of the statistical parameters including central tendency (e.g. means) or other basic estimates (e.g. regression coefficient) AND variation (e.g. standard deviation) or associated estimates of uncertainty (e.g. confidence intervals) |
| ☐ | ☒ | For null hypothesis testing, the test statistic (e.g. $F$, $t$, $r$) with confidence intervals, effect sizes, degrees of freedom and $P$ value noted<br>*Give P values as exact values whenever suitable.* |
| ☒ | ☐ | For Bayesian analysis, information on the choice of priors and Markov chain Monte Carlo settings |
| ☒ | ☐ | For hierarchical and complex designs, identification of the appropriate level for tests and full reporting of outcomes |
| ☒ | ☐ | Estimates of effect sizes (e.g. Cohen's $d$, Pearson's $r$), indicating how they were calculated |

*Our web collection on statistics for biologists contains articles on many of the points above.*

## Software and code

Policy information about availability of computer code

Data collection

TEM (HT7700, HITACH, and JEM-200F)
DLS (Malvern Zetasizer Nano ZS90)
XRD (D8 Advance diffractometer)
UV (UV-3600 PLUS, Shimadzu)
FTIR (Thermo-Nicolet Nexus 670A)
XPS (Thermo Scientific Kα photoelectron spectroscopy)
Raman (Thermos scientific, DXR3xi, 532 nm Filter)
EPR (Bruker EMXnano spectrometer)
VSM (LakeShore7404 spectrometer)
Mott–Schottky plots (CHI760D electrochemical station)
Hydrogen electrode (Unisense, Denmark)
Xenon lamp CHF-XM500)
Illumina NovaSeq 6000 sequencer (CHI BIOTECH Co., Ltd)
Microscope (Nikon, FHEIPSE, Japan)
Image Station (Tanon-5200, China)
Confocal laser scanning microscope (LEICA-SP5 II)
BioTek Synergy HIM microplate reader (CA, USA)
Optical microscope (Olympus BX-41/Q-Color3, Japan)
Fluorescence spectrophotometer (Thermo)

| | |
|---|---|
| | Gas chromatograph（GC, Agilent-7890B） |
| Data analysis | Microscopic images were analyzed by using ImageJ (version 1.51K). Statistical data analyses were performed using GraphPad Prism (version 9). |

For manuscripts utilizing custom algorithms or software that are central to the research but not yet described in published literature, software must be made available to editors and reviewers. We strongly encourage code deposition in a community repository (e.g. GitHub). See the Nature Portfolio guidelines for submitting code & software for further information.

## Data

Policy information about availability of data

All manuscripts must include a data availability statement. This statement should provide the following information, where applicable:

- Accession codes, unique identifiers, or web links for publicly available datasets
- A description of any restrictions on data availability
- For clinical datasets or third party data, please ensure that the statement adheres to our policy

All the data supporting the findings of this study are available within the article and its supplementary information files and from the corresponding author upon reasonable request.

## Human research participants

Policy information about studies involving human research participants and Sex and Gender in Research.

| | |
|---|---|
| Reporting on sex and gender | n/a |
| Population characteristics | n/a |
| Recruitment | n/a |
| Ethics oversight | n/a |

Note that full information on the approval of the study protocol must also be provided in the manuscript.

# Field-specific reporting

Please select the one below that is the best fit for your research. If you are not sure, read the appropriate sections before making your selection.

☒ Life sciences ☐ Behavioural & social sciences ☐ Ecological, evolutionary & environmental sciences

For a reference copy of the document with all sections, see nature.com/documents/nr-reporting-summary-flat.pdf

# Life sciences study design

All studies must disclose on these points even when the disclosure is negative.

| | |
|---|---|
| Sample size | On the premise of ensuring the reliability of the experimental equipment and the sensitivity of the detection method, we ensure the operation standard and stability in the experimental process. When there is no "non-processing factor" that will significantly affect the experimental results, we choose the sample size of n=3 for Figure 3b, n=4 or 5 for Figure 3c, n=4 for Figure 3D, n=3 for Figure 3f, n=3 for Figure 3g, n=4 for Figure 4c, n=3 for Figure 5a. |
| Data exclusions | No data were excluded. |
| Replication | The experiments were replicated or performed independently the same day. We confirm that all attempts at replication were successful. |
| Randomization | In all cell experiments and animal experiments, we randomly assigned the experimental group to compare the different effects under such strict conditions. |
| Blinding | The group allocation of cell and mice was conducted in a blinded manner. Investigators were also blinded to group assignments during data collection and analysis. |

# Reporting for specific materials, systems and methods

We require information from authors about some types of materials, experimental systems and methods used in many studies. Here, indicate whether each material, system or method listed is relevant to your study. If you are not sure if a list item applies to your research, read the appropriate section before selecting a response.

## Materials & experimental systems

| n/a | Involved in the study |
|---|---|
| [ ] | [x] Antibodies |
| [ ] | [x] Eukaryotic cell lines |
| [x] | [ ] Palaeontology and archaeology |
| [ ] | [x] Animals and other organisms |
| [x] | [ ] Clinical data |
| [x] | [ ] Dual use research of concern |

## Methods

| n/a | Involved in the study |
|---|---|
| [x] | [ ] ChIP-seq |
| [x] | [ ] Flow cytometry |
| [x] | [ ] MRI-based neuroimaging |

# Antibodies

Antibodies used

Rabbit monoclonal Anti-RAGE antibody (1:200, abcam, ab216329)
Rabbit polyclonal Anti-AGE antibody (1:200, abcam, ab23722)
Mouse monoclonal Anti-β-Tubulin antibody (1:2000, ABclonal, AC021)
Rabbit monoclonal Anti-GAPDH antibody (1;3000, abcam, ab199553)
Mouse monoclonal Anti-VEGF antibody (1:200, Sant Cruzsc-57496)
Mouse monoclonal Anti-CD31 antibody (1:1000, abcam, ab222783)
Anti-rabbit lgG, HRP-linked Antibody (1:1500, Cell Signaling, 7074)
Anti-mouse lgG, HRP-linked Antibody (1:1500, Cell Signaling, 7076)

Validation

Abcam claims that the Rabbit monoclonal Anti-RAGE antibody (abcam, ab216329) is suitable for mice and IHC.
Abcam claims that the Rabbit polyclonal Anti-AGE antibody (abcam, ab23722) is suitable for mice and IHC.
ABclonal had verified the species and application of the Mouse monoclonal Anti-β-Tubulin antibody (ABclonal, AC021).
Abcam claims that the Rabbit monoclonal Anti-GAPDH antibody (abcam, ab199553) is suitable for mice and WB.
Sant Cruzsc claims that the Mouse monoclonal Anti-VEGF antibody (1:200, Sant Cruzsc-57496) is suitable for mice and WB.
Abcam claims that the Mouse monoclonal Anti-CD31 antibody (abcamab222783) is suitable for mice and WB.

# Eukaryotic cell lines

Policy information about cell lines and Sex and Gender in Research

| | |
|---|---|
| Cell line source(s) | HaCaT, HSF and HMEC-1 cell lines were purchased from Procell Life Science & Technology Co., Ltd. (Wuhan, China). |
| Authentication | None of the cell lines used were authenticated. |
| Mycoplasma contamination | Mycoplasma contamination was not detected. |
| Commonly misidentified lines (See ICLAC register) | No commonly misidentified cell lines were used. |

# Animals and other research organisms

Policy information about studies involving animals; ARRIVE guidelines recommended for reporting animal research, and Sex and Gender in Research

| | |
|---|---|
| Laboratory animals | Mice were housed in groups (four to six) in plastic cages on a 12-hour light/dark cycle. The temperature is kept at about 24 - 26 °C and the humidity is about 60%. |
| Wild animals | No wild animals were used in this study. |
| Reporting on sex | Male C57BL/6J mice were used in this study. |
| Field-collected samples | The study did not involve field-collected samples. |
| Ethics oversight | All animal experiments were carried out under guidelines evaluated and approved by the Ethics Committee of Shenzhen University. |

Note that full information on the approval of the study protocol must also be provided in the manuscript.
