# Supplementary material for: Photocatalytic glucose depletion and hydrogen generation for diabetic wound healing
Source: Nat Commun. 2022 Sep 27;13:5684. doi: 10.1038/s41467-022-33475-7 (PMC9515190; doi:10.1038/s41467-022-33475-7)
Supplement: Supplementary file 3 — Source Data [file 41467_2022_33475_MOESM3_ESM.pdf]

Western blots in Supplementary Figure 31a

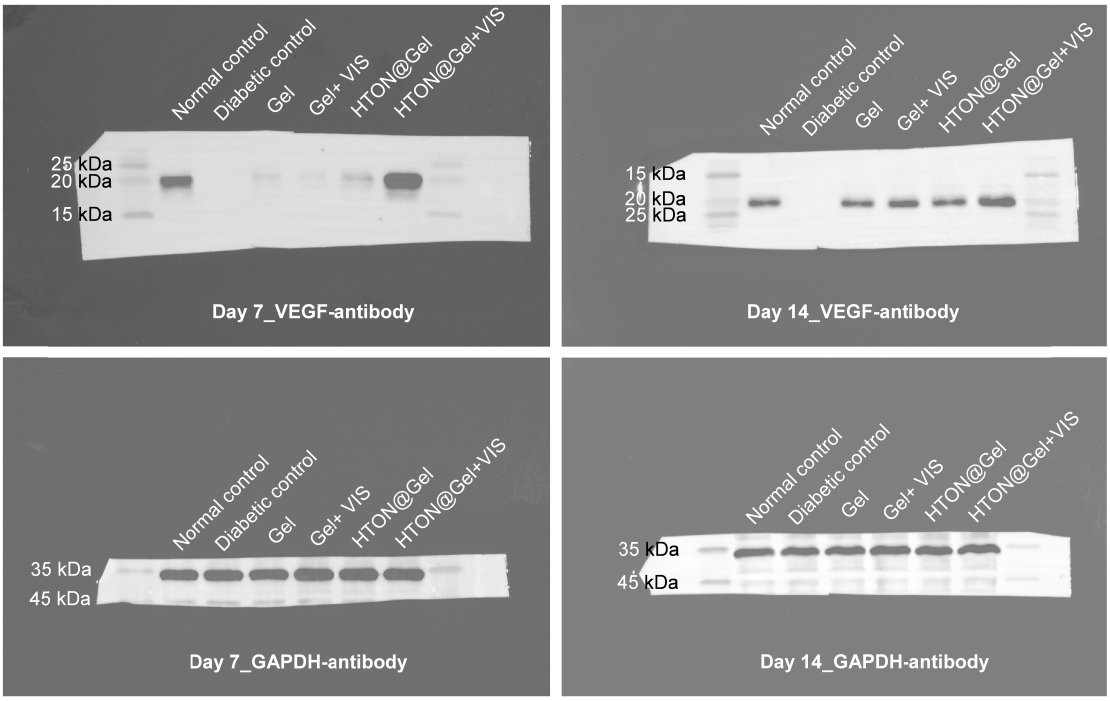

	Normal control	Diabetic control	Gel	Gel+VIS	HTON@Gel	HTON@Gel+VIS
Day 7 VEGF	0.778556694	0.021017657	0.081741421	0.039925941	0.167134096	1.008069683
	0.747093174	0.029682263	0.077157236	0.042378975	0.169849339	0.987554998
Day 14 VEGF	0.601756325	0.012924888	0.548210198	0.570761883	0.550096104	0.896658479
	0.612334912	0.032118842	0.5234593	0.540404497	0.518053782	0.863346246

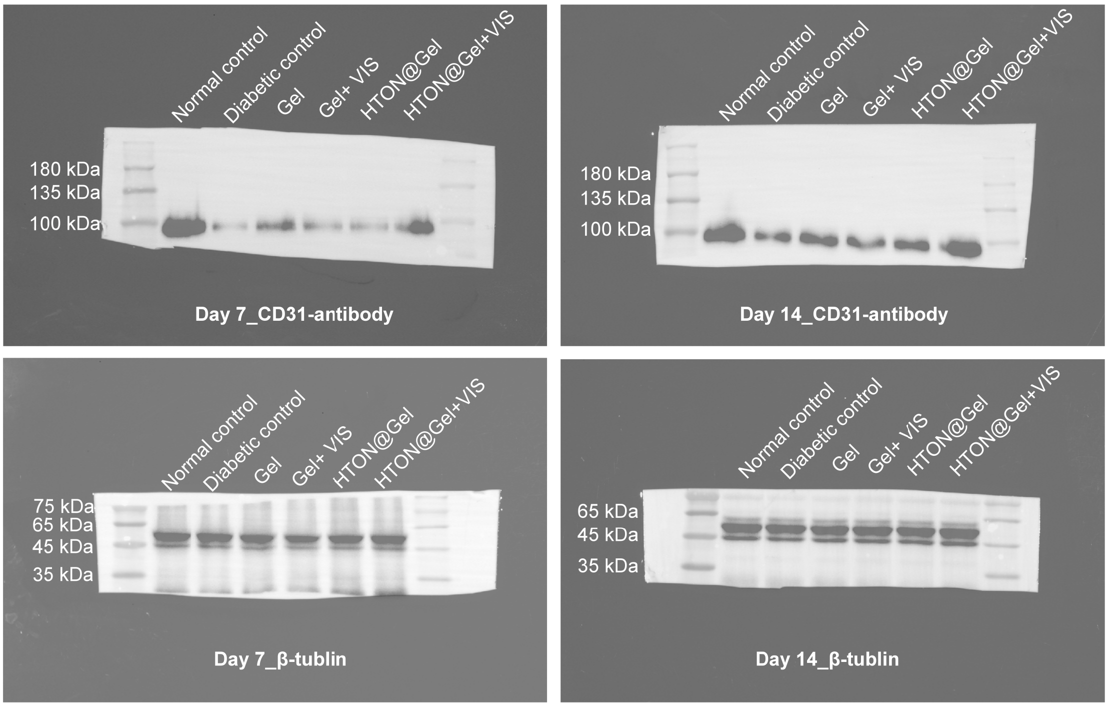

	Normal control	Diabetic control	Gel	Gel+VIS	HTON@Gel	HTON@Gel+VIS
Day 7 CD31	1.117524497	0.122406346	0.424513919	0.276249659	0.178714223	0.663728273
	1.208458347	0.159195756	0.395818265	0.294165564	0.200508128	0.713014174
Day 14 CD31	0.860533858	0.394848817	0.580605517	0.384910279	0.472739751	0.745761931
	0.964954806	0.418589344	0.580605517	0.40134071	0.501626795	0.800027407
